# Microglia Maintain Homeostatic Conditions in the Developing Rostral Migratory Stream

**DOI:** 10.1523/ENEURO.0197-22.2023

**Published:** 2023-02-07

**Authors:** Sarah J. Meller, Lexie Hernandez, Eduardo Martin-Lopez, Zachary A. Kloos, Teresa Liberia, Charles A. Greer

**Affiliations:** 1 Department of Neuroscience, Yale University School of Medicine, New Haven, CT 06520; 2 Department of Neurosurgery, Yale University School of Medicine, New Haven, CT 06520; 3 The Interdepartmental Neuroscience Graduate Program, Yale University School of Medicine, New Haven, CT 06520; 4 Department of Microbial Pathogenesis, Yale University School of Medicine, New Haven, CT 06520

**Keywords:** development, microglia, rostral migratory stream

## Abstract

Microglia invade the neuroblast migratory corridor of the rostral migratory stream (RMS) early in development. The early postnatal RMS does not yet have the dense astrocyte and vascular scaffold that helps propel forward migrating neuroblasts, which led us to consider whether microglia help regulate conditions permissive to neuroblast migration in the RMS. GFP-labeled microglia in *CX3CR-1^GFP/+^* mice assemble primarily along the outer borders of the RMS during the first postnatal week, where they exhibit predominantly an ameboid morphology and associate with migrating neuroblasts. Microglia ablation for 3 d postnatally does not impact the density of pulse labeled BrdU+ neuroblasts nor the distance migrated by tdTomato electroporated neuroblasts in the RMS. However, microglia wrap DsRed-labeled neuroblasts in the RMS of P7 *CX3CR-1^GFP/+^;DCX^DsRed/+^* mice and express the markers CD68, CLEC7A, MERTK, and IGF-1, suggesting active regulation in the developing RMS. Microglia depletion for 14 d postnatally further induced an accumulation of CC3+ DCX+ apoptotic neuroblasts in the RMS, a wider RMS and extended patency of the lateral ventricle extension in the olfactory bulb. These findings illustrate the importance of microglia in maintaining a healthy neuroblast population and an environment permissive to neuroblast migration in the early postnatal RMS.

## Visual Abstract

**Figure:**
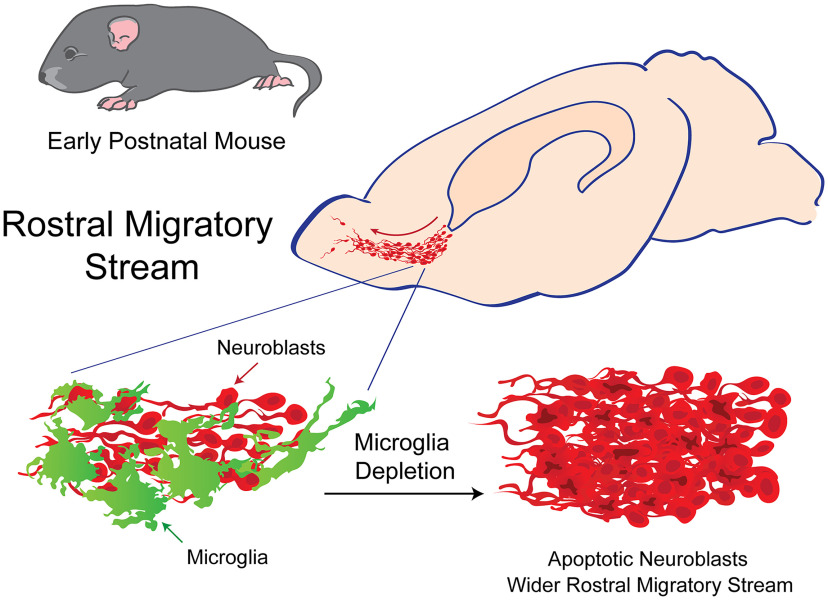

## Significance Statement

Microglia are brain-resident immune cells responsible for maintaining homeostatic conditions necessary for normal neurodevelopment. Microglia phagocytosis is essential during critical periods of development to clear the results of over-exuberant neurogenesis, while microglia-expressed growth factors provide trophic support. Microglial activities may be of special relevance to the olfactory system, which is unique for both a prolonged period of neurogenesis and increased immunologic threat. This work examines how microglia maintain homeostatic conditions in the neuroblast migratory corridor of the rostral migratory stream (RMS) in the olfactory system during early postnatal development. Our findings illustrate the importance of microglia in promoting an environment that allows for effective neuroblast migration in the RMS and has implications for neuroblast migration elsewhere in the nervous system.

## Introduction

Microglia are brain-resident immune cells that maintain homeostatic conditions necessary for normal neurodevelopment and orchestrate responses to environmental insults (Pierre et al., 2017; Tay et al., 2017; Hammond et al., 2018; Lenz and Nelson, 2018; Li and Barres, 2018; Ferro et al., 2021). The importance of microglia in normal neurodevelopment was recently underscored by the case of a child born without microglia because of a homozygous mutation in colony stimulating factor 1 receptor (CSF1R), which is necessary to maintain a microglia population (Nandi et al., 2012). This child presented with agenesis of the corpus callosum, ventriculomegaly, and heterotopias (Oosterhof et al., 2019). These mirrored the findings of a *Csf1r* knock-out mouse model, which also demonstrated ventricular enlargement, disrupted olfactory bulb (OB) architecture and olfactory deficits (Erblich et al., 2011). Microglia functions may be of special relevance to the olfactory system, which is unique in both its vulnerability to environmental insults as well as an extended period of neurogenesis and neuronal migration.

The rostral migratory stream (RMS) is an important neuroblast migratory corridor in the olfactory system. In mice, neuroblasts are continuously generated in the subventricular zone (SVZ) and migrate in chains down the RMS to the OB (Lois and Alvarez-Buylla, 1994; Ponti et al., 2013; Bordiuk et al., 2014). Upon reaching the OB, neuroblasts differentiate into periglomerular and granule cell interneurons and integrate into preexisting circuits (Lledo and Saghatelyan, 2005; Whitman and Greer, 2009). Neuronal migration via the RMS is also active in the human fetus and continues for several months postnatally (Sanai et al., 2011; Wang et al., 2011). The blood vessel scaffold and dense astrocyte tube that wraps the RMS and supports migrating neuroblasts (Bovetti et al., 2007; Snapyan et al., 2009; Whitman et al., 2009; Grade et al., 2013; Gengatharan et al., 2016; Fujioka et al., 2017; Todd et al., 2017) does not assemble until several weeks after birth (Law et al., 1999; Peretto et al., 2005; Nie et al., 2010; Bozoyan et al., 2012), leaving a functional void that may be filled by microglia.

Sustained neurogenesis and neuroblast migration in the RMS may require “quality assurance” measures via microglia phagocytosis. Microglia phagocytosis is essential during critical periods of development for addressing over-exuberant neurogenesis (Cunningham et al., 2013; Barger et al., 2019) and synaptogenesis (Tremblay et al., 2010; Paolicelli et al., 2011; Gunner et al., 2019; Mallya et al., 2019; Scott-Hewitt et al., 2020). In the RMS of adult animals apoptotic cells accumulated following microglia depletion in the SVZ (Ribeiro Xavier et al., 2015) and selective depletion of MERTK in microglia (Fourgeaud et al., 2016), a receptor tyrosine kinase involved in initiating phagocytosis (Rosin et al., 2021), implicating a role for microglia phagocytosis in regulating the neuroblast population. Microglial activities may be especially crucial during the early postnatal period, when there is prolific migration of neuroblasts before the formation of the blood vessel and astrocyte scaffold.

During early postnatal periods a specialized microglia subtype may mediate neurodevelopment. Single-cell sequencing studies identified a microglia subtype with an ameboid morphology and phagocytic markers, including lysosomal-associated membrane protein 1 (Lamp1), CD68, and CLEC7A, in developing white matter tracts during the first postnatal week (Hagemeyer et al., 2017; Hammond et al., 2019; Li et al., 2019). These cells are involved in phagocytosis in developing white matter tracts (Li et al., 2019), but also express the trophic factors insulin-like growth factor 1 (IGF-1) and osteopontin (SPP1; Hagemeyer et al., 2017; Hammond et al., 2019; Li et al., 2019) and are implicated in supporting myelination (Hagemeyer et al., 2017; Wlodarczyk et al., 2017) and axons (Ueno et al., 2013). Intriguingly, postnatal microglia expressing the phagocytic marker CLEC7A are also seen in the RMS (Li et al., 2019), suggesting that a similar microglia subset may be involved in phagocytosis or trophic support in the developing RMS.

We investigated whether microglia regulate the population of migrating neuroblasts in the RMS during the early postnatal period. We examined the distribution and morphology of RMS microglia and employed depletion strategies to gain insights into their function. We found that ameboid microglia distribute along the borders of the early postnatal RMS before the formation of the blood vessel scaffold and astrocyte tube. Microglia depletion did not impact the migratory capacity of neuroblasts but did compromise the homeostasis of the RMS, causing an accumulation of neuroblasts and apoptotic cells that broadened the domain of the early postnatal RMS. Microglia therefore appear critical for maintaining the homeostasis of the RMS neuroblast population and migratory environment.

## Materials and Methods

### Animals

Experiments investigating microglia morphology and distribution were conducted with B6.129P2(Cg)-Cx3cr1^tm1Litt^/J (The Jackson Laboratory stock #005582) mice, which express EGFP in microglia under control of the *Cx3cr1* locus (Jung et al., 2000). The mice were maintained as homozygous colonies and crossed with C57BL/6J mice to produce heterozygous *CX3CR1^GFP/+^* mice for experiments. To examine the interactions between microglia and neuroblasts, *CX3CR-1^GFP/GFP^* mice were crossed with C57BL/6J-Tg(Dcx-DsRed)14Qlu/J (a gift from Angeliki Louvi), which express the red fluorescent protein variant DsRed under the control of the doublecortin (*Dcx*) locus (X. Wang et al., 2007). This allowed the generation of *CX3CR-1^GFP/+^;DCX^DsRed/+^* transgenic mice.

To selectively deplete microglia *in vivo,* B6.129P2(Cg)-*Cx3cr1^tm2.1(cre/ERT2)Litt/^*WganJ (a gift from Arie Kaffman), which express a Cre-ERT2 fusion protein under the *CX3CR1* promoter (Parkhurst et al., 2013), were crossed with *Gt(ROSA)26Sor^tm1(DTA)Jpmb^*/J (a gift from Arie Kaffman), which express a *loxP*-flocked STOP cassette before the diphtheria toxin fragment A (DTA; Ivanova et al., 2005). Homozygous *Cx3cr1^CreER/CreER^* were crossed with heterozygous *ROSA26^eGFP-DTA/+^* mice to create *Cx3cr1^CreER/+^; ROSA26^eGFP-DTA/+^* and *Cx3cr1^CreER/+^; ROSA26^+/+^* control littermates. Tamoxifen injection induces the expression of the toxic DTA subunit in microglia in *Cx3cr1^CreER/+^; ROSA26^eGFP-DTA/+^* mice.

All experiments randomly included both male and female mice, although sex comparisons were not pursued. Mice were housed with a 12 h light/dark cycle with access to standard food and water *ad libitum*. All animal care and use were performed in accordance with the Institutional Animal Care and Use Committee (IACUC) Yale animal care committee’s regulations.

### Electroporation

To label individual migrating neuroblasts, stem cells lining the lateral wall of the lateral ventricle were electroporated with a pCAG-tdTomato at P0 and killed at P2, P4, P7, P14. The pCAG-tdTomato plasmid used for electroporation was a gift from Angelique Bordey (Addgene plasmid #83029; http://n2t.net/addgene:83029), which was maintained in *Escherichia coli* strains stored at −80°C as 10% glycerol:LB stock. P0 mice were anesthetized with hypothermia before injection with tdTomato plasmid. The plasmid solution was injected into one of the brain lateral ventricles using a Picospritzer (General Valve Corporation). Electroporations were made by delivering five pulses of 35 V using a pair of gold tweezers (Genepaddles-542, Harvard Apparatus) connected to an ECM 830 electroporator (BTX Harvard Apparatus).

### Liposome injection

Microglia were depleted in perinatal mice by the injection of clodronate-filled liposomes (Clodrosome) at postnatal day (P0). Injection of clodronate-filled liposomes and their subsequent phagocytosis by microglia induces cell death, reflected by a dramatic decrease in the number of microglia 3 d later (Cunningham et al., 2013). A total of 2 µl of Clodrosomes or the control PBS-filled liposomes (Encapsome) were injected into the cerebral lateral ventricles of CX3CR-1^GFP/+^ mice bilaterally using a Picospritzer (General Valve Corporation; Table 1). The effect of microglia knock-down was evaluated 3 d later by immunohistochemistry and confocal microscopy. To demonstrate the phagocytic capacity of microglia throughout the RMS, fluorescent liposomes containing the fluorescent dye Dil (Fluoroliposome) were injected into the cerebral lateral ventricles of P1 CX3CR-1^GFP/+^ mice and killed at P4 (Table 1). P0 postnatal mice were anesthetized with hypothermia before injection.

**Table 1:** Liposomes used in the study

Liposomes	Company (catalog #)
Standard Macrophage Depletion kit (Clodrosome + Encapsome)	Encapsula NanoSciences (CLD-8901)
Fluoroliposome-DiI	Encapsula NanoSciences (CLD-8911)

### BrdU injections

To analyze the density of neuroblasts migrating in the RMS, *CX3CR-1^GFP/+^;DCX^DsRed/+^* mice were injected at P0 with 25 mg/kg of the thymidine analog BrdU (BD PharMingen) to label newborn neuroblasts and killed at P3. To investigate the number of neuroblasts that reach the glomerular layer following microglia depletion, *CX3CR-1^GFP/+^* mice were injected with 50 mg/kg of BrdU at P1 and killed at P4.

### Tamoxifen injections

**Figure 1.**
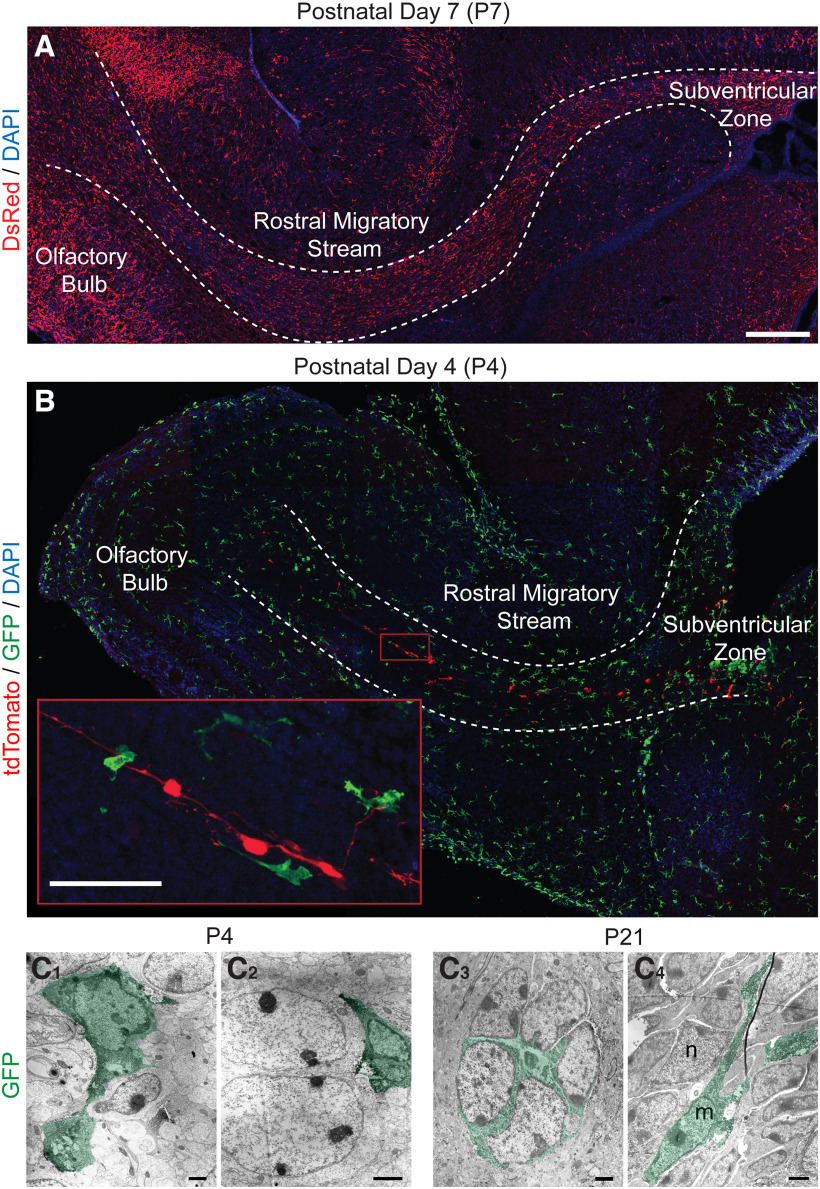
Microglia line the rostral migratory stream (RMS) and contact migrating neuroblasts during early postnatal development. ***A***, Sagittal section showing neuroblasts (red) in a P7 *DCX^DsRed/+^* mouse. There is active migration of neuroblasts from the subventricular zone (SVZ) to the olfactory bulb (OB) via the RMS in the first postnatal week. Scale bar = 250 µm. ***B***, Sagittal section showing microglia (green) in the SVZ, RMS, and OB in a P4 *CX3CR-1^GFP/+^* mouse. To identify individual migrating neuroblasts (red), the SVZ was electroporated with a tdTomato plasmid at P0 and killed at P4. Microglia line the borders of the RMS and associate with migrating neuroblasts within the core. Scale bar = 50 µm. ***C***, Ultrathin sections showing GFP-immunopositive microglia soma and processes at the electron microscope level in *CX3CR-1^GFP/+^* mice at P8 and P21. Microglia are pseudo-colored in green. ***C_1_***, ***C_2_***, Ameboid microglia at P4 contact migrating neuroblasts. ***C_1_***, A microglial cell engaged in phagocytosis, as shown by the engulfment of material within the extended phagocytic cup. ***C_3_***, ***C_4_***, Microglia processes contact migrating neuroblasts at P21. ***C_3_***, A microglia between a cluster of migrating neuroblasts. m, microglial cell. n, neuroblast. Scale bar = 2 µm.

To deplete microglia in *Cx3cr1^CreER/+^; ROSA26^eGFP-DTA/+^* mice, 30 μg/g of tamoxifen (TMX; Sigma T5648) was injected at P0. TMX induces Cre-mediated recombination at loxP sites and removal of the terminal STOP codon sequence, enabling expression of the DTA fragment (Fig. 1). Mice were either killed at P3 or injected with TMX every subsequent 3 d to maintain microglia depletion before P14. *Cx3cr1^CreER/+^; ROSA26^+/+^* littermates served as controls.

### Tissue processing

For immunofluorescence, mice were deeply anesthetized with an overdose of Euthasol (Virbac) and perfused transcardially in 0.1 m PBS, pH 7.4 with 1 unit/ml heparin, followed by 4% paraformaldehyde (PFA) (JT Baker) in PBS. Animals were decapitated and the brains carefully removed from the skull. The brains were postfixed in 4% PFA at 4°C overnight. Following PBS washes, brains were transferred to a 30% sucrose and kept at 4°C for 3–4 d for cryoprotection. Brains were then embedded in OCT (optimal cutting temperature) compound (Thermo Fisher Scientific); 25-µm sections were serially collected in both coronal and sagittal planes using a Reichert Frigocut Cryostat E-2800. Sections were frozen at −20°C until use.

For electron microscopy, the mice were perfused with 4% PFA and 0.75% glutaraldehyde, followed by postfixation in the same fixative for 4 h. Brains were rinsed in PBS overnight and cut on a vibratome (50 μm).

### Immunohistochemistry

Sections were thawed at 37°C and treated for antigen retrieval with 0.01 m citrate buffer at pH 6 and 68°C for 30 min. They were incubated in a blocking solution of PBS supplemented with 0.1% Triton X-100 (Sigma-Aldrich) and 0.2% bovine serum albumin (BSA; Sigma-Aldrich) for 1 h at room temperature. Sections were incubated in a mixture of primary antibodies diluted in blocking solution at 4°C overnight, followed by incubation with secondary antibodies diluted in blocking solution at 1:400 for 2 h at room temperature (Table 2). Nuclei were counterstained by incubating the sections with 1 µg/ml DAPI for 10 min (Invitrogen). Sections were washed with PBS after each step. The sections finally mounted with Mowiol 4–88 (Sigma-Aldrich).

**Table 2.** Primary and secondary antibodies used in the study

Primary antibodies				
Anti	Host	Isotype	Dilution	Company (catalog #)
GFP	Chicken	Polyclonal	1:500	Abcam (Ab13970)
GFAP	Mouse	Monoclonal IgG1	1:400	Sigma-Aldrich (G3893)
CD31	Mouse	Monoclonal IgG1	1:50	Abcam (ab9498)
Iba1	Rabbit	Polyclonal	1:200	Wako (019–19741)
CC3	Rabbit	Polyclonal	1:400	Cell Signaling (9661)
MERTK	Rat	Monoclonal IgG2a	1:100	Invitrogen (14-5751-82)
CLEC7A	Rat	Monoclonal IgG2a	1:30	Invivogen (mabg-mdect)
CD68	Rat	Monoclonal IgG2a	1:200	Biorad (FA-11)
DCX	Guinea pig	Polyclonal	1:400	Sigma-Aldrich (AB2253)

Secondary antibodies				
Anti	Host	Isotype	Dilution	Company

Chicken	Donkey	A488	1:400	The Jackson Laboratory
Chicken	Goat	Biotin	1:400	Abcam
Mouse	Goat	A647	1:400	Molecular Probes
Rabbit	Donkey	A488	1:400	Molecular Probes
Rabbit	Donkey	A555	1:400	Molecular Probes
Rat	Donkey	A647	1:400	The Jackson Laboratory
Guinea pig	Goat	A555	1:400	Molecular Probes

### Electron microscopy

Tissue was incubated with 2% BSA (Sigma) in PBS for 30 min to block nonspecific binding sites. The tissue was incubated in a chicken polyclonal anti-GFP antibody (Abcam) in blocking buffer for 4 d at room temperature. Tissue was then incubated in biotin-conjugated goat anti-chicken IgY secondary antibodies for 1 h at room temperature. Sections were incubated with the ABC reagent (Vector) for 1 h, followed by a DAB peroxidase reaction. Sections were immediately processed for electron microscopy.

Stained tissue was postfixed with 4% osmium tetroxide for 1 h, dehydrated through graded alcohols, and polymerized in Epon between glass slides and coverslips coated previously with Liquid Release Agent (Electron Microscopy Sciences). Areas of interest were selected based on the DAB labeling and re-embedded in Epon. These re-embedded sections were cut using an ultramicrotome in 70-nm-thick ultrathin sections. These 70-nm sections were examined with a JEOL transmission electron microscope and photographed at primary magnifications of 3000–4000×.

### Fluorescence *in situ* hybridization

To produce an RNA probe antisense to *Igf1* mRNA, forward primer 5′- TGAGCTCAGCCAGTTCGTGTGTGGACCGAGGGG-3′(containing a BlpI restriction site at its 5′ end) and reverse primer 5′-TGAAGATCTTAATACGACTCACTATAGGCGGCTCTTTAGAGGCAGGGACTAGG-3′ (containing a BglII restriction site, followed by a T7 promoter sequence, at its 5′ end) were used to amplify a 646 nucleotide region of the *Igf1* coding sequence (nucleotides 485–1130, spanning the junctions between exons 2 and 3, 3 and 4, and 4 and 5) from cDNA prepared from OB tissue isolated from eight-week-old wild-type (WT) CD-1 mice. Following sequential digestion with BlpI and BglII, the resulting amplicon was cloned into the corresponding restriction sites of plasmid pET-28a such that the *Igf1* cassette was flanked by a T7 terminator at its 3′ end. Using the resulting construct as template and an Atto 647N RNA Labeling kit (Jena Bioscience), fluorescent RNA probes were generated by in vitro transcription according to the manufacturer’s recommendations.

### Confocal imaging and processing

All images were taken using a Zeiss LSM800 confocal with either a 10× or 40× objective and images processed with Fiji (ImageJ) software. When creating tiled images of entire sagittal sections for illustrative purposes, images were taken with the 10× objective at 27-µm z-stacks (four slices at 6.75-µm step distance). After creating a maximum projection of each channel, a “rolling ball” algorithm was applied to correct for uneven illumination.

Cell density analysis was performed on confocal images obtained with the 10× objective. Z-stacks were 27 µm in depth (four slices at 6.75-µm step distance.) The rostral migratory stream (RMS) was first isolated on a 16-bit maximum projection of a 638.9 × 638.9-µm image of DAPI nuclear staining with a freehand drawing tool. The area of this drawn region of interest (ROI) was measured later to determine the density of stained cells and area ratio of stained processes. The ROI was drawn on the maximum projection of the DAPI channel to prevent bias for incorporating stained cells of interest. A mask of the drawn ROI was then applied to the corresponding channels stained with antibodies against GFP, GFAP, Iba1, BrdU, and CC3 to quantify stained objects within this ROI.

There was increased noise in the GFP and DsRed channels, and thus we applied an additional “despeckle” function, which removes salt-and-pepper noise. Salt-and-pepper noise reduction was achieved in the CC3 channel with a “median” filter of pixel radius 1. BrdU staining was punctate, so to blur punctate staining and isolate stained nuclei a “Gaussian blur” function with a pixel σ of 2 was applied to the BrdU maximum projection. To segment stained pixels from background we used the Fiji automatic Otsu threshold function, except for segmenting Iba1 and CC3 stained objects, for which we used the Fiji automatic Triangle threshold function. A gray scale attribute filter with an opening function of area minimum of 100 pixels and connectivity of 8 was further applied using the MorphoLibJ integrated library and plugin (Legland et al., 2016) to isolate the cell bodies of GFP-stained and Iba1-stained microglia and BrdU-stained neuroblasts. A gray scale attribute filter with an opening function of area minimum of 15 pixels and connectivity of 8 was applied to isolate the cell bodies of DsRed-stained neuroblasts, and of an area minimum of 50 pixels and connectivity of 8 to isolate CC3-stained cells. After making a mask of the images, the density of stained cell bodies was assessed by counting the number of cells with the “Analyze Particles” function and normalizing this number to the area of the drawn ROI for the layer. GFAP staining was quantified by the percent area stained within the drawn ROI.

The density of CC3-stained cells in the RMS descent following 3 and 14 d of microglia depletion was performed on confocal images obtained with the 40× objective. Z-stacks were 18 µm in depth (15 slices at 1.2-µm step distance.) A “rolling ball” algorithm with a radius of 50 pixels was applied to subtract background and salt and pepper noise reduction was achieved with a “median” filter of pixel radius 3. After creating a maximum projection of the CC3 channel, stained pixels were segmented from background using the Fiji automatic Otsu threshold function. To fill in the nucleus we applied an opening filter of a diamond radius 2 using the MorphoLibJ integrated library and plugin (Legland et al., 2016). All segmented CC3-stained nuclei were then manually compared with the Z-stack of merged DCX, DAPI and CC3 channels to assess whether DCX staining surrounded the isolated CC3+ DAPI+ nucleus. Finally, the density of CC3+ DCX+ and CC3+ DCX− stained nuclei were assessed by normalizing the number to the area of the drawn ROI of the RMS from a maximum projection of the DAPI channel. The total number of CC3+ stained nuclei was verified using the “Analyze Particles” function.

We employed the ImageJ plugin FracLac to assess the morphology of individual binarized microglial and neuroblast cells (Young and Morrison, 2018). 40× confocal images were taken of GFP+ microglia and tdTomato+ neuroblasts in the RMS elbow, and maximum projections were made of 24 µm (10 slices at 1.2-µm step distance) to collapse the whole cell and its processes. Maximum projections were obtained for four sections of the RMS elbow per animal, with every cell that had all of its processes contained within the image analyzed. For the GFP channel, a “Despeckle” function was first used to correct for noise, followed by a “remove outliers” function to target bright outliers with a pixel radius of 2 and threshold of 50. The GFP+ cell was isolated from background using the “default” automatic threshold and binarized. The “close” plugin was then used to connect two pixels if they were separated by up to two pixels. To enhance the small features of neuroblasts and remove background in the tdTomato channel, a “difference of Gaussians” was applied to the max projection. The tdTomato+ cell was isolated from background using the “triangle” automatic threshold and binarized before performing fractal analysis.

To perform fractal analysis on an individual cell in the image, a rectangle selection of 100 pixels of both height and width was applied for the ROI of all cells, to ensure that all the cells have the same scale. While fractal shapes are scale-independent, FracLac for ImageJ is dependent on scale, and thus the ROI needs to be consistently sized throughout the data collection (Karperien, 1999; Young and Morrison, 2018). Using the matching maximum projection photomicrograph as a reference, the paintbrush tool was used to isolate the cell of interest by removing adjacent cell processes and connecting fragmented processes (Young and Morrison, 2018). The isolated cell was then converted to an outline using the “outline” function. Fractal analysis data are gathered via box counting, in which a series of grids of decreasing caliber were systematically laid over an image and the number of boxes containing a pixel counted. The fractal dimension (D_B_) is the relationship between how a pattern’s detail (N) changes with the scale (ε), or resolution, at which the image is considered (Karperien et al., 2013). Fractal analysis was done with the Box Counting in FracLac plugin, and data were collected with four box counting orientations (Num G = 4). Lacunarity (Λ) is also calculating using the box counting method and is a measure of cell heterogeneity; cells with low lacunarity are homogenous or rotationally invariant (Karperien et al., 2013). Additional cell shape measurements were generated using the “convex hull” (a polygon that encloses all pixels of the binary cell) and “bounding circle” (the minimum circle that can enclose all pixels.) The span ratio is the ratio of the longest length over the longest length of the convex hull (Morrison et al., 2017). Density is the ratio of the area of cell divided by the area of its convex hull. A full list and explanation of FracLac output data are explained in the FracLac for ImageJ manual (Karperien, 1999). The calculations to obtain the fractal dimension (D_B_), lacunarity (Λ), span ratio and density are best explained in the reference guide provided for FracLac for ImageJ (http://rsb.info.nih.gov/ij/plugins/fraclac/FLHelp/Introduction.htm).

We further used FracLac to evaluate the lacunarity of a max projection of DCX-stained processes in the RMS as a measure of the space occupied by neuroblast processes following 14 d of microglia depletion. Confocal images were obtained with the 40× objective. Z-stacks were 18 µm in depth (15 slices at 1.2-µm step distance.) After obtaining a maximum projection, DCX-stained processes were segmented from background with a “Otsu” automatic threshold. A mask of the drawn ROI obtained using a freehand drawing tool on the maximum projection of the DAPI channel was applied and DCX-stained processes outside the RMS subtracted from the image. Fractal analysis was done with the Box Counting in FracLac plugin, and data were collected with four box counting orientations (Num G = 4). Lacunarity (Λ) values are an indicator of the “gappiness” between stained processes, with high lacunarity suggesting greater gaps and variation between stained processes (Karperien et al., 2013).

3D rendering to show the accumulation of fluoroliposomes in microglia was performed using IMARIS software using the “Blend” mode (Bitplane, Imaris Viewer; https://imaris.oxinst.com/imaris-viewer).

### Statistical analysis

All measures across different sections within an animal from the same RMS region were averaged to yield one sample replicate for statistical analysis. Statistical analysis was performed with GraphPad Prism 9.2 for windows (GraphPad Software Inc). The data were analyzed with a one-way ANOVA followed by Bonferroni’s *post hoc* test or a Student’s *t* test as appropriate. *p <0.05, **p < 0.01, ***p < 0.001, ****p < 0.0001. Data are shown as the mean ± SEM (Standard error of the mean). Specific *p* values for each experiment are shown in the statistical table (Table 3).

**Table 3:** Summary of statistical analysis employed in each experiment

Experiment	Test	*P*-value	Statistical value	*Post hoc* test	Pair-wise comparison	*P*-value
Figure 3*D_1_* microglia cell density	One-way ANOVA	0.3100	*F*_(3,10)_ = 2.386	Bonferroni’s correction		
Figure 3*D_2_* GFAP^+^ area	One-way ANOVA	<0.0001	*F*_(3,10)_ = 1.635	Bonferroni’s correction	P4 vs P56 P7 vs P56 P21 vs P56	0.0003 0.0001 0.0093
Figure 4*B_1_* microglia fractal dimension	One-way ANOVA	0.0015	*F*_(5,16)_ = 6.757	Bonferroni’s correction	P2 vs P21 P2 vs P56 P4 vs P56	0.0113 0.0021 0.0292
Figure 4*D* microglia density	One-way ANOVA	<0.0001	*F*_(5,16)_ = 13.92	Bonferroni’s correction	P2 vs P4 P2 vs P7 P2 vs P14 P2 vs P21 P2 vs P56	0.0135 0.0024 <0.0001 0.0006 <0.0001
Figure 5*B_1_* microglia cell density-RMS start	Student’s *t* test	<0.0001	*F*_(4,4)_ = 1.742			
Figure 5*B_1_* microglia cell density-RMS descent	Student’s *t* test	0.0005	*F*_(4,4)_ = 2.890			
Figure 5*B_2_* neuroblast cell density-RMS start	Student’s *t* test	0.0072	*F*_(4,4)_ = 1.850			
Figure 5*B_2_* neuroblast cell density-RMS descent	Student’s *t* test	0.9883	*F*_(4,4)_ = 1.436			
Figure 5*B_3_* BrdU cell density-RMS start	Student’s *t* test	0.9651	*F*_(4,4)_ = 1.417			
Figure 5*B_3_* BrdU cell density-RMS start	Student’s *t* test	0.3559	*F*_(4,4)_ = 1.311			
Figure 6*C_1_* microglia cell density-RMS start	Student’s *t* test	<0.0001	*F*_(2,3)_ = 28.69			
Figure 6*C_1_* microglia cell density-RMS descent	Student’s *t* test	<0.0001	*F*_(2,3)_ = 885.1			
Figure 6*C_2_* neuroblast cell density-RMS start	Student’s *t* test	0.8665	*F*_(2,3)_ = 12.85			
Figure 6*C_2_* neuroblast cell density-RMS descent	Student’s *t* test	0.7748	*F*_(2,3)_ = 4.314			
Figure 6*C_2_* neuroblast cell density-RMS horizontal limb	Student’s *t* test	0.4449	*F*_(2,3)_ = 4.314			
Figure 6*C_3_* neuroblast density ratio- descent/start	Student’s *t* test	0.4940	*F*_(2,3)_ = 2.541			
Figure 6*C_3_* neuroblast density ratio-horizontal limb/descent	Student’s *t* test	0.9312	*F*_(2,3)_ = 1.813			
Figure 6*F_1_* neuroblast fractal dimension	Student’s *t* test	0.7133	*F*_(2,2)_ = 1.008			
Figure 6*F_2_* neuroblast lacunarity	Student’s *t* test	0.6047	*F*_(2,2)_ = 1.662			
Figure 6*F_3_* neuroblast density	Student’s *t* test	0.8467	*F*_(2,2)_ = 19056			
Figure 6*F_4_* neuroblast span ratio	Student’s *t* test	0.7007	*F*_(2,2)_ = 500.8			
Experiment in Figure 9 microglia cell density-RMS start	Student’s *t* test	<0.0001	*F*_(3,3)_ = 162.5			
Experiment in Figure. 9 microglia cell density-RMS descent	Student’s *t* test	<0.0001	*F*_(3,3)_ = 123.9			
Figure 9*B_2_* CC3+ density-RMS descent	Two-way ANOVA	Marker = 0.0099 Genotype = 0.0168 Interaction = 0.029	Marker *F*_(1,5)_ = 16.33 Genotype *F*_(1,5)_ = 12.43 Interaction *F*_(1,5)_ = 19.199	Bonferroni’s correction	WT vs DTA: CC3+/DCX+ WT vs DTA: CC3+/DCX−	0.0018 ns
Figure 10*B_1_* CC3+ density-RMS descent	Two-way ANOVA	Marker = 0.0212 Genotype = 0.0238 Interaction = 0.0281	Marker *F*_(1,10)_ = 7.45 Genotype *F*_(1,10)_ = 7.083 Interaction *F*_(1,10)_ = 6.584	Bonferroni’s correction	WT vs DTA: CC3+/DCX+ WT vs DTA: CC3+/DCX−	0.0029 ns
Figure 10*B_2_* RMS width-RMS descent	Student’s *t* test	0.0035	*F*_(5,5)_ = 2.335			
Figure 10*B_3_* DCX stain lacunarity	Student’s *t* test	0.0494	*F*_(5,5)_ = 1.339			

n.s.: not significant.

## Results

### Ameboid microglia are present in the developing RMS before the formation of the astrocyte and vascular scaffold

There is active migration of neuroblasts from the SVZ to the OB through the RMS during the first postnatal week, as shown by dense staining of DsRed+ neuroblasts in the RMS of *DCX^DsRed/+^* mice (Fig. 1*A*). During the first postnatal week microglia line the RMS along its lateral borders (Fig. 1*B*). To examine the interactions between individual microglia and neuroblasts, neuroblasts were electroporated to express tdTomato. Within the RMS, microglia closely appose individual neuroblasts (Fig. 1*B*, inset). This close interaction between microglia and neuroblasts within the RMS was confirmed with electron microscopy (Fig. 1*C_1_–C_4_*), which showed microglia and their processes (green) intimately associated with migrating neuroblasts. We identified neuroblasts based on the following ultrastructural features: multiple nuclei within the nucleus, a dark cytoplasm with many free ribosomes, and the presence of extracellular spaces between neuroblasts (Doetsch et al., 1997; Matsumoto et al., 2019). In the biotin-labeled microglia distinctive features included the accumulation of chromatin on the inner membrane of the nucleus, the presence of lipofuscin granules in the cytoplasm and their comparatively small size relative to neuroblasts within the RMS (Fig. 1*C_1_–C_4_*).

**Figure 2.**
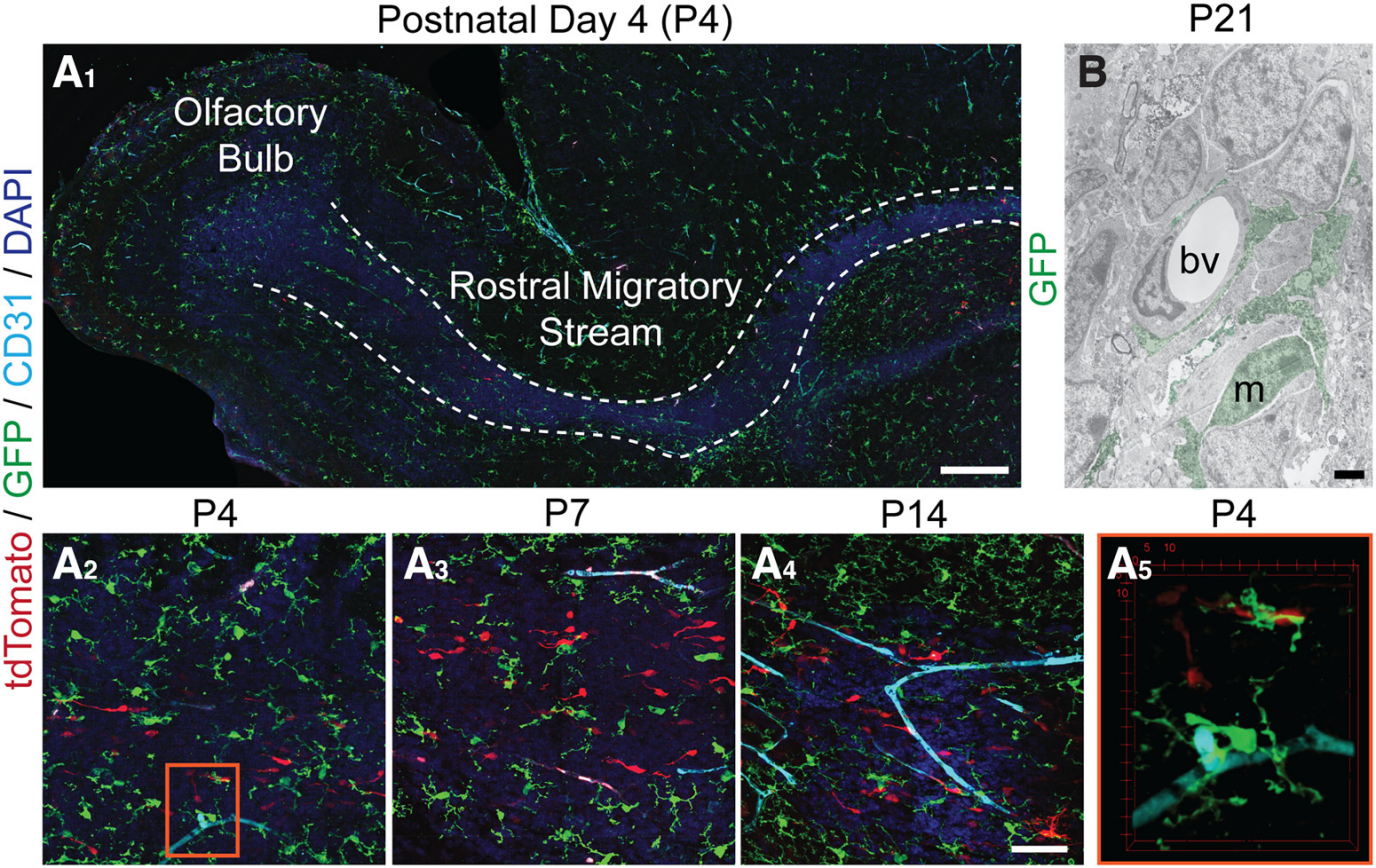
Microglia are present in the rostral migratory stream (RMS) before the formation of the vascular scaffold. ***A***, Sagittal sections of the RMS from *CX3CR-1^GFP/+^* mice electroporated with a tomato plasmid at P0 and killed at ages P4, P7, and P14. Migrating neuroblasts are depicted with tomato, microglia with GFP, and vasculature with CD31. ***A_1_***, The RMS region was defined by increased density of DAPI nuclear staining, as shown by the white dashed outline. While there is CD31-labeled vasculature in the parenchyma at P4, there is not the stereotypic vascular wrapping of the RMS observed in adulthood. Scale bar = 250 µm. ***A_2_–A_4_***, Microglia closely associate with migrating neuroblasts in the RMS at P4, P7, and P14. Note that the majority of migrating neuroblasts are not in close vicinity of the developing vasculature during the first postnatal week. Scale bar = 50 µm. ***A_5_***, The orange outlined inset depicts a volume rendering of microglia interacting with vasculature and the soma of a migrating neuroblast at the RMS elbow at P4. ***B***, Ultrathin electron micrograph sections showing GFP-immunopositive microglia in a P21 *CX3CR-1^GFP/+^* mouse contacting an endothelial cell of a blood vessel in the RMS. m, microglial cell. bv, blood vessel. Scale bar = 2 µm.

Microglia distribute along the RMS before the establishment of the vascular and astrocyte scaffold. While CD31-stained vasculature is observed during the first postnatal week, it does not show the characteristic wrapping of the RMS that forms the scaffold during adulthood (Fig. 2*A_1_–A_3_*). Numerous tdTomato-labeled neuroblasts are seen at a distance from the developing vasculature, suggesting that they migrate independently of the vasculature during this period. An increase in CD31-stained blood vessels was observed at P14, with the beginnings of a network of vasculature forming around the RMS at this age (Fig. 2*A_4_*). Microglia closely appose endothelial cells in the RMS at P4 and P21 (Fig. 2*A_5_*,*B*).

**Figure 3.**
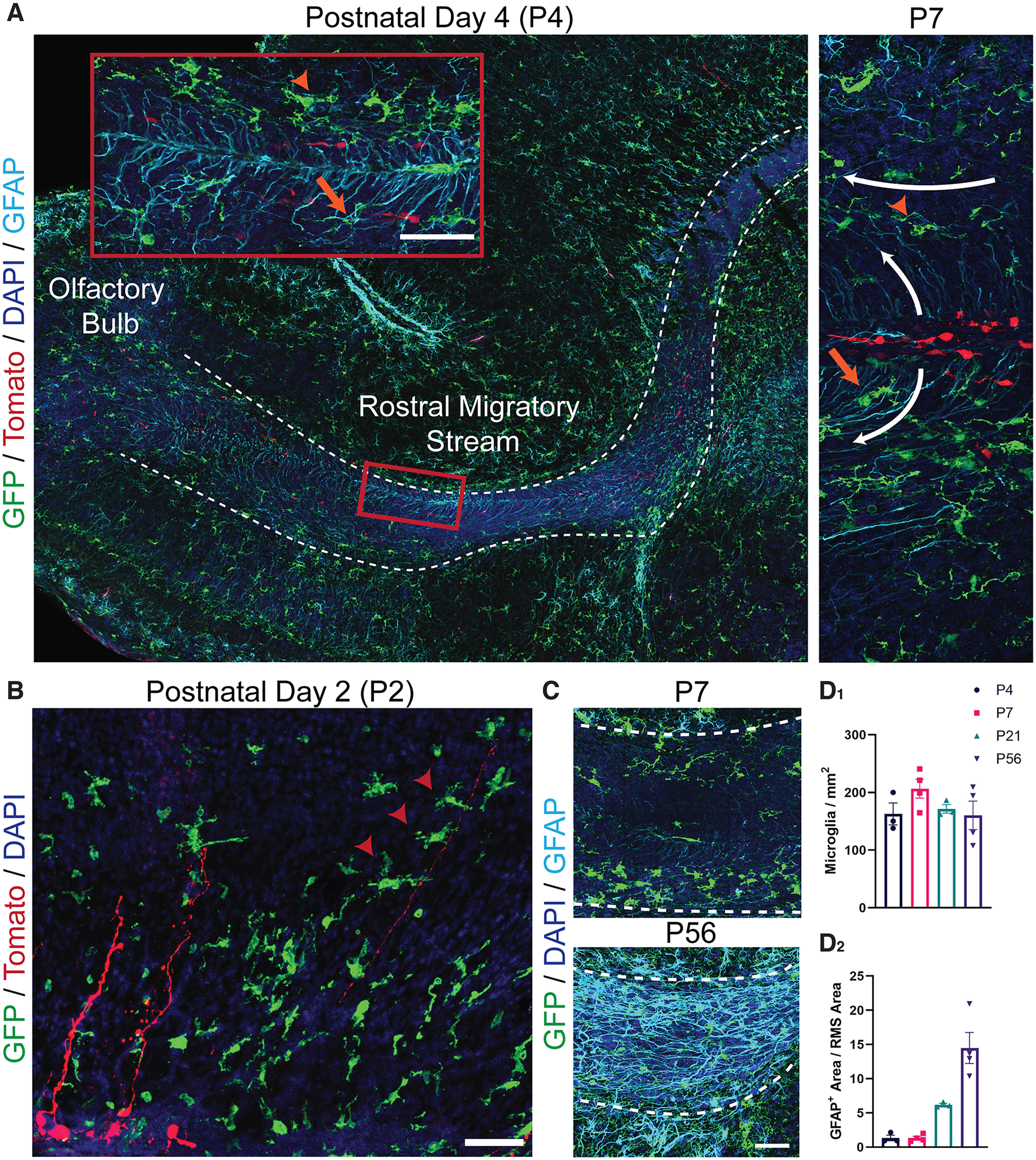
Microglia are present in the rostral migratory stream (RMS) before the formation of the astrocytic tube and interact with radial glia. ***A***, Sagittal sections of the RMS from *CX3CR-1^GFP/+^* mice electroporated with a tomato plasmid at P0 and killed at ages P4 and P7. Migrating neuroblasts are depicted with tomato, microglia with GFP, and radial glia and astrocytes with GFAP. Microglia interact with GFAP-stained radial glial processes tangentially oriented at the border of the RMS (orange arrowheads) and with processes radially distributing within its core (orange arrows). Scale bar = 50 µm. ***B***, Sagittal section of radial glia cell pial fibers extending from the SVZ of a *CX3CR-1^GFP/+^* mouse electroporated with a tomato plasmid at P0 and killed at ages P2. Some microglia interact with pial fibers via a process extension that partially wraps around the fiber in the manner of a “hand-hold” (red arrowheads). Scale bar = 50 µm. ***C***, The RMS elbow from *CX3CR-1^GFP/+^* mice at P7 and P56. Microglia are represented by GFP (green) and radial glia and astrocytes by GFAP (cyan). Scale bar = 50 µm. Microglia are observed predominately at the periphery of the RMS at P7, where they associate with radial glia processes. While the astrocyte tube is not present at P7, the thick septate processes of GFAP-stained astrocytes are present in the RMS by P56. ***D_1_***, There is a dramatic increase in GFAP staining between P4 and P56 in the RMS elbow, reflecting the development of the astrocyte tube. ***D_2_***, Microglia are present in the RMS elbow at similar densities at P4, P7, P21, and P56.

Similarly, an astrocyte tube of thick septate processes that surrounds and infiltrates the RMS is present in the adult mouse (P56) but not during the first postnatal week (P7; Fig. 3*C*). Instead, radial glia stained with glial fibrillary acidic protein (GFAP) radiate out from the RMS core during the first postnatal week (Fig. 3*A*). Microglia organized along the periphery of the RMS during the first postnatal week closely intermingled with the GFAP-stained radial glial processes (Fig. 3*A*). Microglia also contacted the long pial fibers of radial glia with cell bodies in the SVZ (Fig. 3*B*). The gradual formation and thickening of astrocyte processes over development in the RMS is reflected in the significant increase in GFAP staining across the elbow between P4 and P56 (*p* < 0.0001; Fig. 3*D_2_*). By P56 microglia exhibit a more homogeneous distribution across the RMS (Fig. 3*C*), reminiscent of the “tiling” observed in the mature cortex (Nimmerjahn et al., 2005). While the microglia distribution changes over the course of postnatal RMS development, there is no significant difference in the density of GFP+ microglia in the RMS between postnatal ages (Fig. 3*D_2_*).

**Figure 4.**
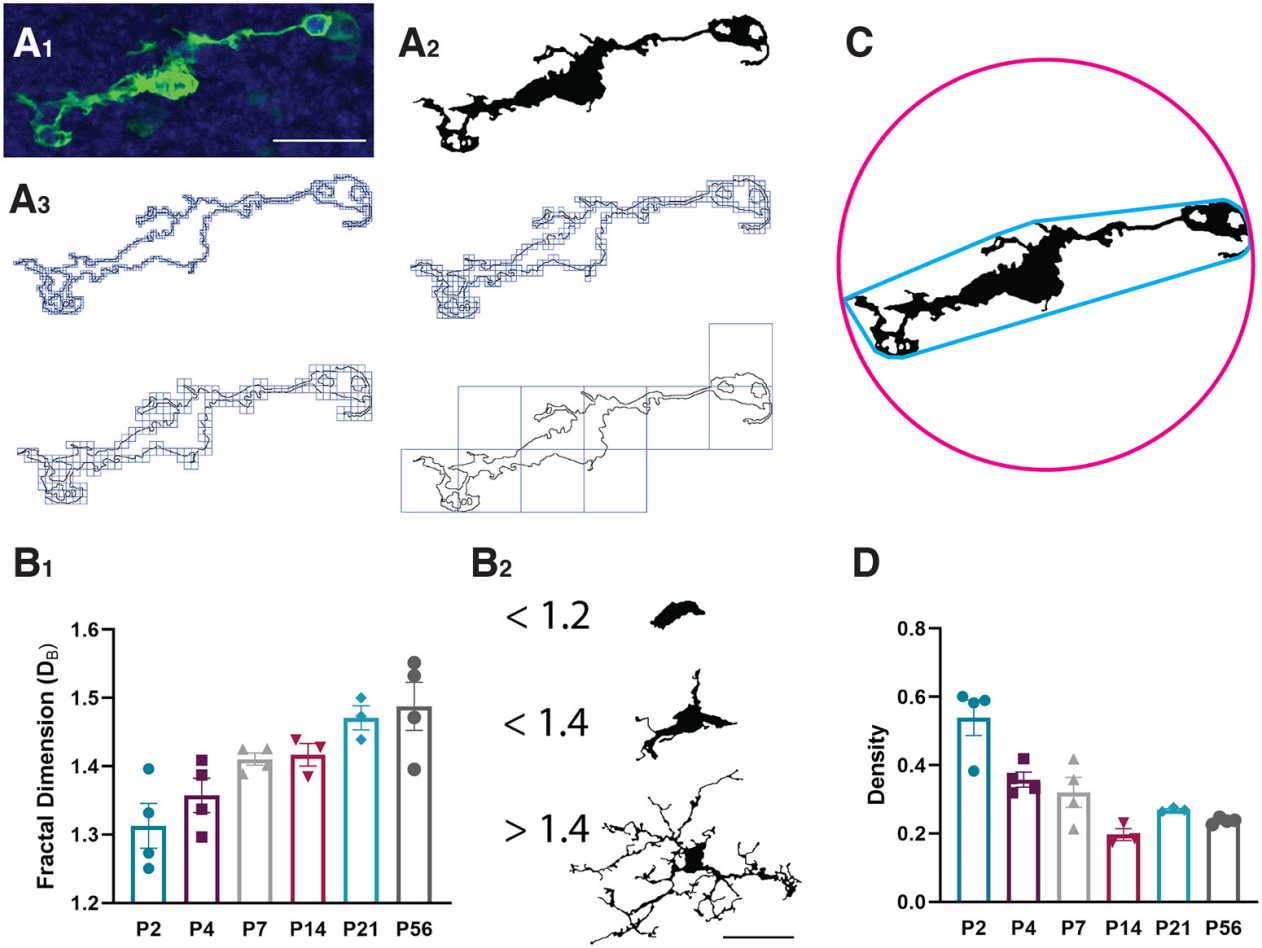
Microglia in the rostral migratory stream exhibit increased average pattern complexity at later postnatal ages. ***A_1_***, A microglial cell from the RMS of a P4 *CX3CR-1^GFP/+^* mouse. Scale bar = 25 µm. ***A_2_***, The microglia from ***A_1_*** segmented and binarized. ***A_3_***, Fractal analysis data are gathered via box counting, in which a series of grids of decreasing caliber were systematically laid over an image and the number of boxes containing a pixel counted. The fractal dimension (D_B_) is the relationship between how a pattern’s detail (N) changes with the scale (ε) at which the image is considered. ***B_1_***, There is significantly increased average pattern complexity at later postnatal ages, as shown by increased measures on the fractal dimension. ***B_2_***, Example microglia shapes and their corresponding fractal dimension D_B._ Scale bar = 25 µm. ***C***, Microglia cell “density” is measured by dividing the area of the cell by the area of its convex hull. The convex hull is in cyan, which is created by connecting a series of straight segments that enclose all the pixels of the binary image. The bounding circle (depicted in magenta) is the smallest circle enclosing all the pixels. ***D***, There is a decrease in the proportion of “dense” and more “ameboid” cells at later postnatal ages. Eight to 20 microglia per animal.

The morphology of microglia in the developing RMS may further give insight into their function. Ameboid microglia are round with short processes and appear to be highly motile and phagocytic (Davalos et al., 2020). Conversely, ramified microglia have long and highly branched processes that continuously sample their local environment (Davalos et al., 2005; Nimmerjahn et al., 2005). Microglia exhibit a greater range of morphologies during the early postnatal period in the RMS, including fewer morphologically complex and more “dense” cells (*p* < 0.0001; Fig. 4). The “dense” microglia observed at P2 and P4 implicate ameboid morphologies in the RMS during the first postnatal week (Fig. 4*D*). There is a decrease in very “dense” and ameboid cells by P14, which is accompanied by an increase in cell complexity (Fig. 4). Microglia in the RMS show a gradual increase in cell process complexity between P2 and P56, as demonstrated by the increase in the average fractal dimension (D_B_) of binarized microglia (*p* < 0.01; Fig. 4*B_1_*). This increase in the complexity of microglia processes reflects an increased ramification. This change in microglia morphology from ameboid to ramified during RMS development could simply be a manifestation of normal microglial cell development. However, it seems plausible that the functional state of microglia adapt in response to the signals from their local tissue environment (Matcovitch-Natan et al., 2016; Li and Barres, 2018), including cues from the emerging astrocyte and vascular scaffolds.

### Neuroblast migratory capacity is independent of microglia in the developing rostral migratory stream

**Figure 5.**
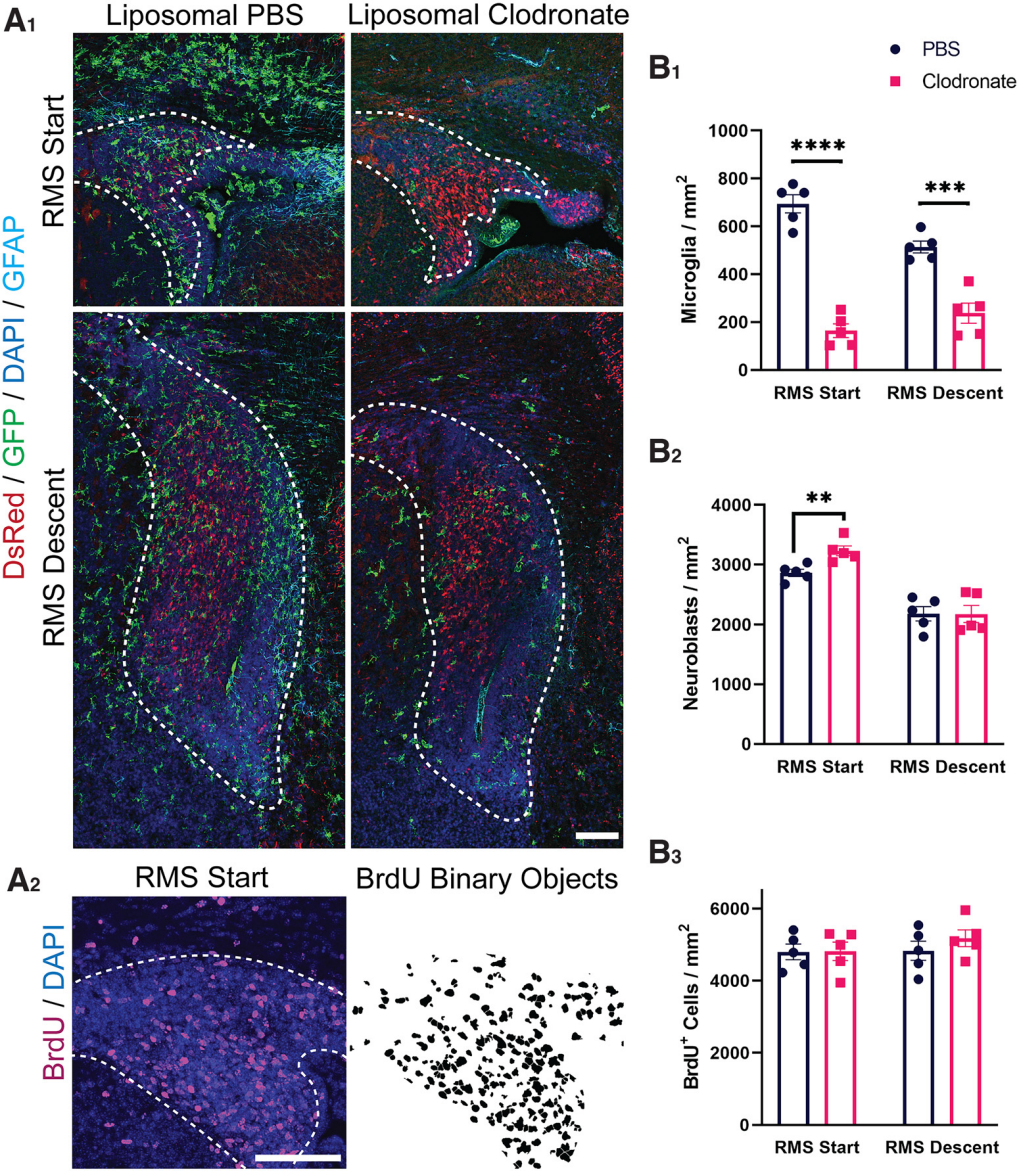
Microglia depletion after liposomal clodronate injection does not impact neuroblast density in the rostral migratory stream (RMS). ***A_1_***, Coronal sections of the RMS start and descent from P3 *CX3CR-1^GFP/+^;DCX^DsRed/+^* littermates following injection with liposomal PBS or liposomal clodronate at P0 and subcutaneous 25 mg/kg BrdU injection 2 h later. The RMS region was defined by increased density of DAPI nuclear staining, as shown by the white dashed outline. Scale bar = 100 μm. ***A_2_***, GFP-stained, DsRed-stained, and BrdU-stained cells were segmented and binarized with Fiji software. Scale bar = 100 μm. ***B_1_***, Three days after injection of liposomal clodronate, there was a significant decrease in microglia density in both the RMS start and RMS descent. ***B_2_***, Microglia depletion was not accompanied by a corresponding decrease in the density of migrating neuroblasts in the RMS descent, as shown by the density of DsRed-labeled cells in this region. There was a significant increase in the density of DsRed-labeled cells in the RMS start following liposomal clodronate treatment, potentially reflecting a decrease in microglia phagocytosis of neural stem cells in the subventricular zone. ***B_3_***, There was no significant difference in the density of newly generated BrdU-labeled neuroblasts in either the RMS start or descent between liposomal PBS and liposomal clodronate treated littermates. **p < 0.01, ***p < 0.001, ****p < 0.0001.

The presence of microglia in the RMS during the first postnatal week prompted the hypothesis that microglia may facilitate neuroblast migration in the absence of the vascular and astrocyte scaffold. We first investigated whether microglia depletion affects the density of neuroblasts migrating down the RMS; neuroblast clumping within the RMS following microglia depletion would implicate a lack of forward migration. Microglia depletion was conducted in *CX3CR-1^GFP/+^;DCX^DsRed/+^* transgenic mice to allow identification of microglia (GFP) and neuroblasts (DsRed; Fig. 5*A_1_*). To further analyze the density of migrating neuroblasts, 25 mg/kg of BrdU was injected at P0 to label actively diving neuroblasts (Fig. 5*A_2_*). Microglia depletion was initiated 2 h later with an injection of clodronate-filled liposomes in the cerebral lateral ventricles. PBS-filled liposomes injected in littermates served as a control. Mice were killed at P3 to determine the density of BrdU-labeled and DsRed-labeled neuroblasts in the RMS start and descent following microglia depletion. There was a significant reduction in the density of microglia in both the RMS start and RMS descent in mice injected with liposomal clodronate as compared with littermates injected with liposomal PBS (*p* < 0.001; Fig. 5*B_1_*). However, microglia depletion was not accompanied by a difference in the density of either DsRed-labeled or BrdU-labeled neuroblasts in the RMS descent (*p* = n.s.; Fig. 5*B_2_*,*B_3_*). While there was a significant increase in the density of DsRed-labeled cells in the RMS start following liposomal clodronate treatment (*p* < 0.01; Fig. 5*B_2_*), there was no significant difference in the density of BrdU-labeled neuroblasts in the RMS start (Fig. 5*B_3_*). The absence of difference in BrdU labeling suggests that neurogenesis was not significantly altered following transient microglia depletion. The increase in DsRed staining may reflect a decrease in microglia phagocytosis of neural stem cells in the SVZ, which was not captured by the temporally sparse BrdU labeling of newly born neuroblasts. The similar density of neuroblasts migrating down the RMS descent following liposomal clodronate treatment indicates that neuroblasts are not stalled in their migration toward the OB in the absence of microglia.

**Figure 6.**
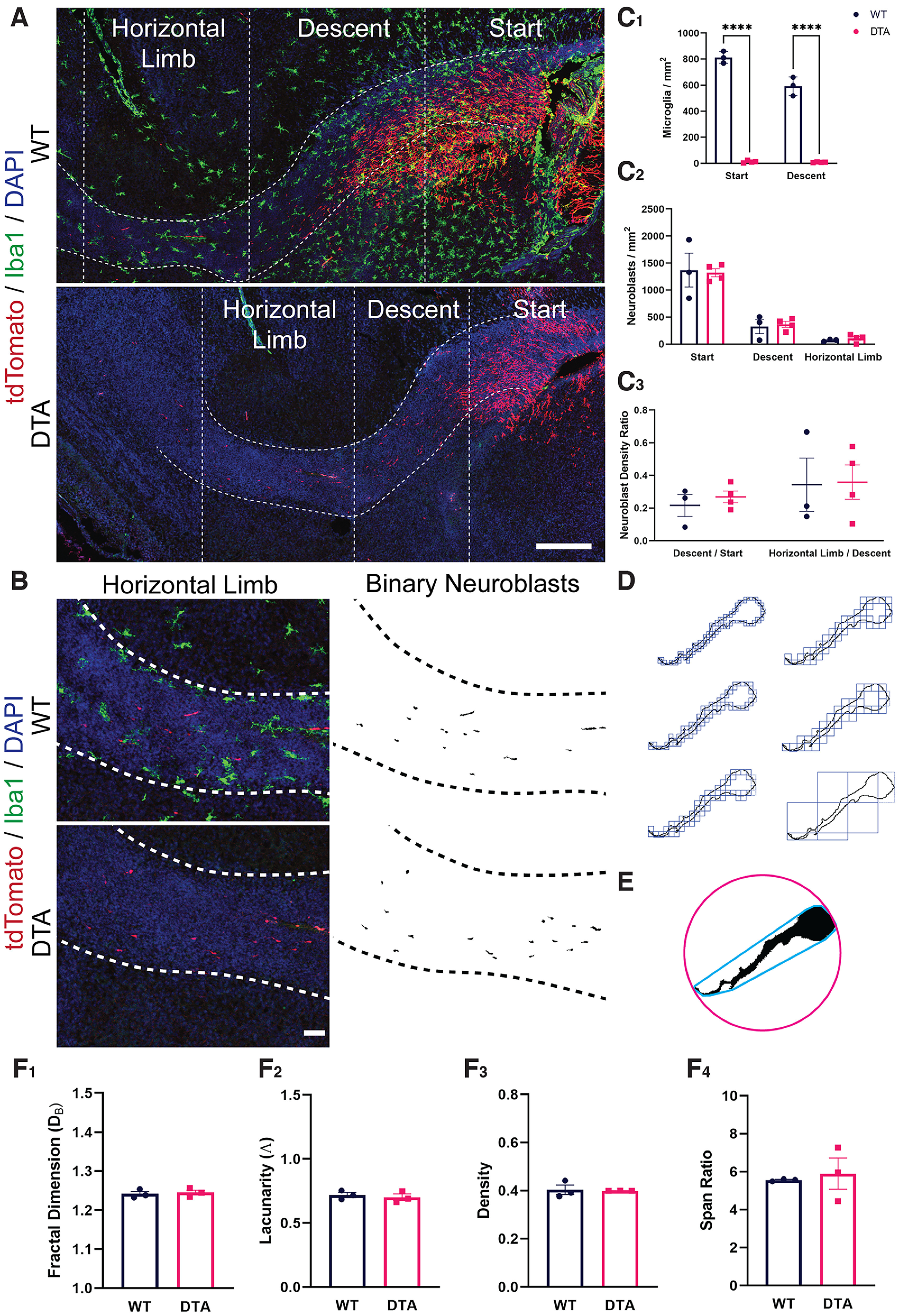
Microglia depletion does not impact the migratory capacity of neuroblasts in the rostral migratory stream (RMS). ***A***, Sagittal sections of the RMS following intraperitoneal tamoxifen injection in *Cx3cr1^CreER/+^; ROSA26^+/+^* (top) and in *Cx3cr1^CreER/+^; ROSA26^eGFP-DTA/+^* (bottom) littermates at P0 and killing at P3. Migrating neuroblasts (red) were labeled by electroporation of a tdTomato plasmid in the subventricular zone (SVZ) at P0. Microglia line the rostral migratory stream (RMS) and contact migrating neuroblasts within it at P3 (top). Scale bar = 250 μm. ***B***, tdTomato-labeled neuroblasts were segmented and binarized with Fiji software. The RMS region was defined by increased density of DAPI nuclear staining, as shown by the white dashed outline. Scale bar = 100 μm. ***C***, There was a significant decrease in the density of Iba1-labeled microglia in the RMS following tamoxifen injection in DTA mice as compared with WT littermates. However, this was not accompanied by a difference in the density of tdTomato-labeled neuroblasts in the RMS start, descent, or horizontal limb. There was also no difference in the ratio of neuroblasts that reached the RMS descent/RMS start, or the ratio of neuroblasts that reached the horizontal limb/the number that were still traveling the descent. ***D***, A segmented and binarized neuroblast. Fractal analysis data are gathered via box counting, in which a series of grids of decreasing caliber were systematically laid over an image and the number of boxes containing a pixel counted. ***E***, A binarized neuroblast showing the convex hull used for morphology analysis. The convex hull is in cyan, which is created by connecting a series of straight segments that enclose all the pixels of the binary image. The bounding circle (depicted in magenta) is the smallest circle enclosing all the pixels. ***F_1_***, There was no significant difference in neuroblast pattern complexity following microglia depletion, as shown by similar measures on the fractal dimension (D_B_). ***F_2_***, There was no significant difference in the heterogeneity of binarized neuroblast cells following microglia depletion, as shown by measures of lacunarity (Λ). ***F_3_***, There was no difference in the “density” of neuroblast morphology following microglia depletion. Density is measured by dividing the area of a cell by the area of its convex hull ***F_4_***, There was no difference in the “span ratio” of neuroblasts in the RMS following microglia ablation. The span ratio is the ratio of the major over minor axes of the convex hull. Nine to 13 neuroblasts per animal. ****p < 0.0001.

To further examine whether microglia promote neuroblast migration we asked whether microglia depletion impacts the distance migrated by individual neuroblasts in the RMS. To accomplish this we used an alternative model of inducible microglia depletion. Homozygous *Cx3cr1^CreER/CreER^* were crossed with heterozygous *ROSA26^eGFP-DTA/+^* mice to create *Cx3cr1^CreER/+^; ROSA26^eGFP-DTA/+^* and *Cx3cr1^CreER/+^; ROSA26^+/+^* littermates. Following tamoxifen injection, the toxic DTA subunit is expressed in *Cx3cr1^CreER/+^; ROSA26^eGFP-DTA/+^* mice, leading to the ablation of microglia. To label individual neuroblasts and evaluate the distance traveled, we electroporated a tdTomato plasmid into the SVZ at P0. We injected tamoxifen intraperitoneal 2 h later to selectively deplete microglia in those littermates that express the toxic DTA subunit. Littermates that did not express the DTA subunit served as controls. We killed the mice at P3 and assessed the density of tdTomato-labeled neuroblasts in the RMS start, descent and horizontal limb (Fig. 6*A–C*) and their morphology (Fig. 6*D*,*E*) following microglia depletion.

Tamoxifen injection at P0 significantly depleted microglia 3 d later in both the RMS start and RMS descent (*p* < 0.0001; Fig. 6*C_1_*). However, this was not accompanied by a difference in the density of neuroblasts in the RMS start, descent, or horizontal limb following microglia depletion in *Cx3cr1^CreER/+^; ROSA26^eGFP-DTA/+^* as compared with *Cx3cr1^CreER/+^; ROSA26^+/+^* littermates (p = n.s.; Fig. 6*C_2_*). There was also no difference in the ratio of tdTomato-labeled neuroblasts that reached the RMS descent over those in the RMS start, or neuroblasts that reached the horizontal limb compared with those still in the RMS descent for each animal (p = n.s.; Fig. 6*C_3_*). The successful migration of neuroblasts to the more distal segments of the RMS following microglia depletion suggests that microglia are not necessary for neuroblast migration in the RMS. We next asked whether microglia depletion influenced neuroblast morphology, potentially hinting at more subtle disorganization of neuroblast migratory patterns (Fig. 6*D*,*E*). However, there was no significant difference in neuroblast pattern complexity (p = n.s.; Fig. 6*F_1_*), heterogeneity (p = n.s.; Fig. 6*F_2_*), density (p = n.s.; Fig. 6*F_3_*) or span ratio (p = n.s.; Fig. 6*F_4_*) following microglia ablation. The similar morphologies of neuroblasts traversing the RMS further indicates that microglia ablation does not impact the migratory capacity of neuroblasts.

### Microglia mediate the homeostasis of the developing rostral migratory stream

The microglia cell distribution and ameboid morphology pattern in the early postnatal RMS suggests that microglia may monitor the RMS environment and apply trophic support or “quality assurance” measures through phagocytosis. Single-cell microglia analysis identified a subset with a distinct gene expression profile in the first postnatal week in the developing corpus callosum that express the phagocytic markers CD68 and CLEC7A and trophic factors IGF-1 and SPP1 (Hagemeyer et al., 2017; Hammond et al., 2019; Li et al., 2019). Microglial expression of receptor tyrosine kinase MERTK, which is important in the adult RMS (Fourgeaud et al., 2016), is also generally upregulated on the surface of embryonic microglia compared with the adult (Rosin et al., 2021).

**Figure 7.**
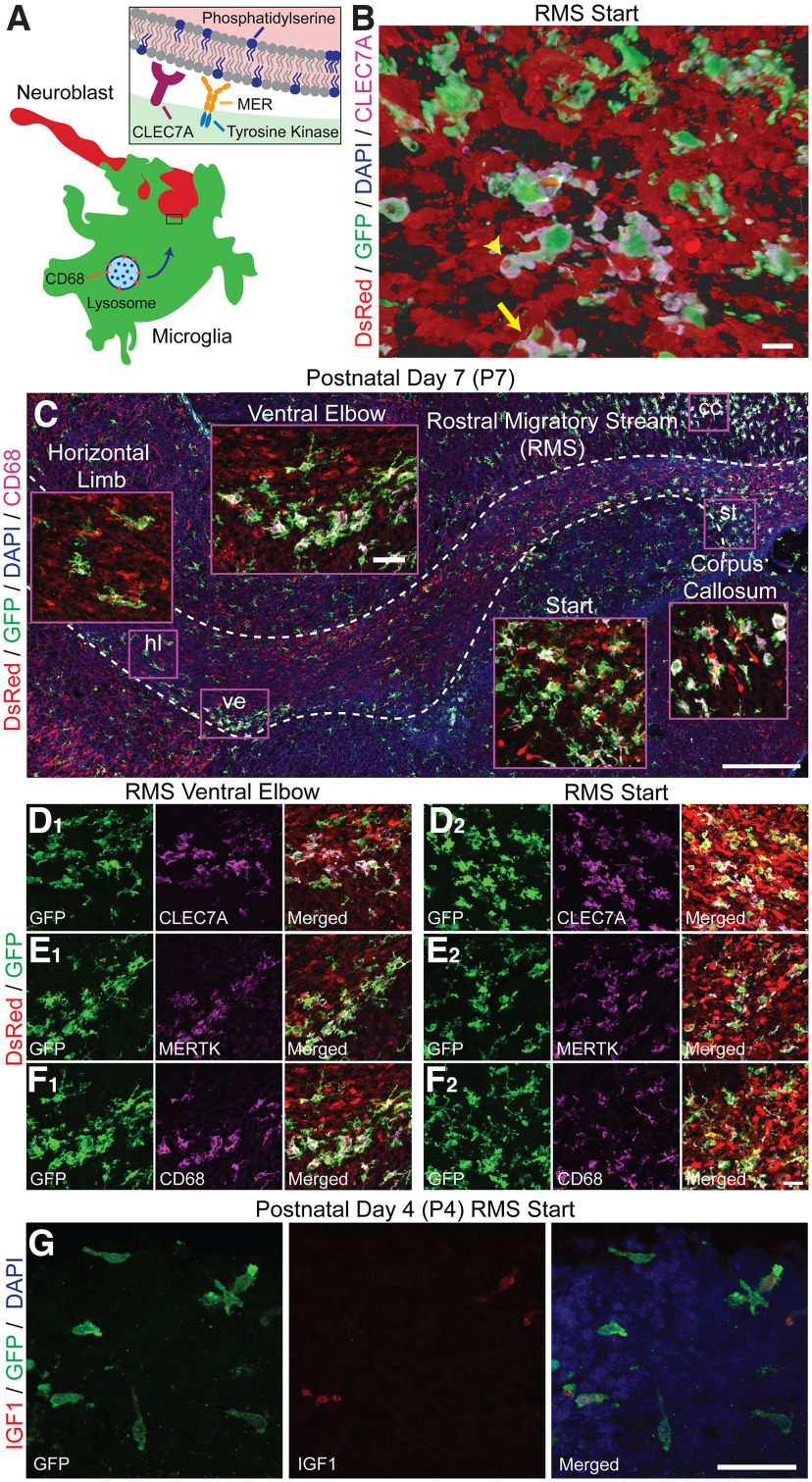
Specialized microglia subtype in the postnatal rostral migratory stream (RMS). ***A***, Illustration of microglia phagocytic markers. CD68 is a lysosome-associated transmembrane glycoprotein that is upregulated with phagocytosis. CLEC7A, or Dectin-1, is a transmembrane pattern-recognition receptor that triggers phagocytosis and the release of reactive oxygen species (ROS). MERTK is a member of the TAM (Tyro3, Axl, and Mertk) family of receptor tyrosine kinases that recognize soluble bridging proteins that bind externalized phosphatidylserine, including GAS6 and protein S. ***B***, 3D volume rendering of GFP+/CLEC7A+ microglia and DsRed+ neuroblasts in the RMS start of a P7 mouse with Imaris software. The yellow arrow shows an example of a GFP+/CLEC7A+ microglial cell “wrapping” a DsRed+ neuroblast. The yellow arrowhead indicates DsRed-labeled material within a microglia phagocytic cup. Scale bar = 10 µm. ***C***, Sagittal section showing the interactions between microglia (green) and neuroblasts (red) in the RMS (dashed white outline) of a P7 *CX3CR-1^GFP/+^;DCX^DsRed/+^* mouse. Microglia exhibited the phagocytic marker CD68 while “wrapping” neuroblasts in the RMS. Microglia CD68 expression was marked at the start of the RMS and at the ventral border of the elbow. CD68+ microglia also “hugged” neuroblasts radially migrating from the subventricular zone through the corpus callosum and start of the RMS. cc, corpus callosum; st, start; ve, ventral elbow. Scale bar = 250 µm. Scale bar of magnified inset = 25 µm. Microglia expressed the phagocytic markers CLEC7A (***D***), MERTK (***E***), and CD68 (***F***) while wrapping neuroblasts at the start of the RMS (***C_2_***, ***D_2_***, ***F_2_***) and at the ventral border of the RMS elbow (***C_1_***, ***D_1_***, ***F_1_***). Scale bar = 25 µm. ***G***, Microglia expressing high transcript levels of the trophic factor *Igf1* neighbor microglia with no *Igf1* expression at the RMS start of a P4 *CX3CR-1^GFP/+^* mouse. Scale bar = 25 µm.

We capitalized on these recent findings to ask whether microglia in the RMS the phagocytic markers CLEC7A, MERTK, CD68 (Fig. 7*A*) and trophic factor IGF-1 during early postnatal development. Microglia expressed phagocytic markers while “hugging” neuroblasts in the RMS of P7 *CX3CR-1^GFP/+^;DCX^DsRed/+^* mice and contained DsRed material within phagocytic cups (Fig. 7*B*). Microglia also expressed phagocytic markers while “hugging” neuroblasts in the corpus callosum of P7 *CX3CR-1^GFP/+^;DCX^DsRed/+^* mice (Fig. 9*B*), consistent with previous studies (Li et al., 2019). Clusters of microglia expressing the phagocytic markers CD68, CLEC7A, and MERTK were also found wrapping DsRed+ neuroblasts in the RMS start and ventral border of the RMS elbow (Fig. 7*D–F*). Occasional microglia expressing phagocytic markers were also found to wrap migrating neuroblasts in the core of the RMS descent and horizontal limb (Fig. 7*C*). Microglia in the RMS start of P4 *CX3CR-1^GFP/+^* mice expressed high transcript levels of *Igf1*; however, there was heterogeneity of expression, with microglia expressing high transcript levels of *Igf1* immediately adjacent to microglia with no appreciable expression (Fig. 7*G*).

**Figure 8.**
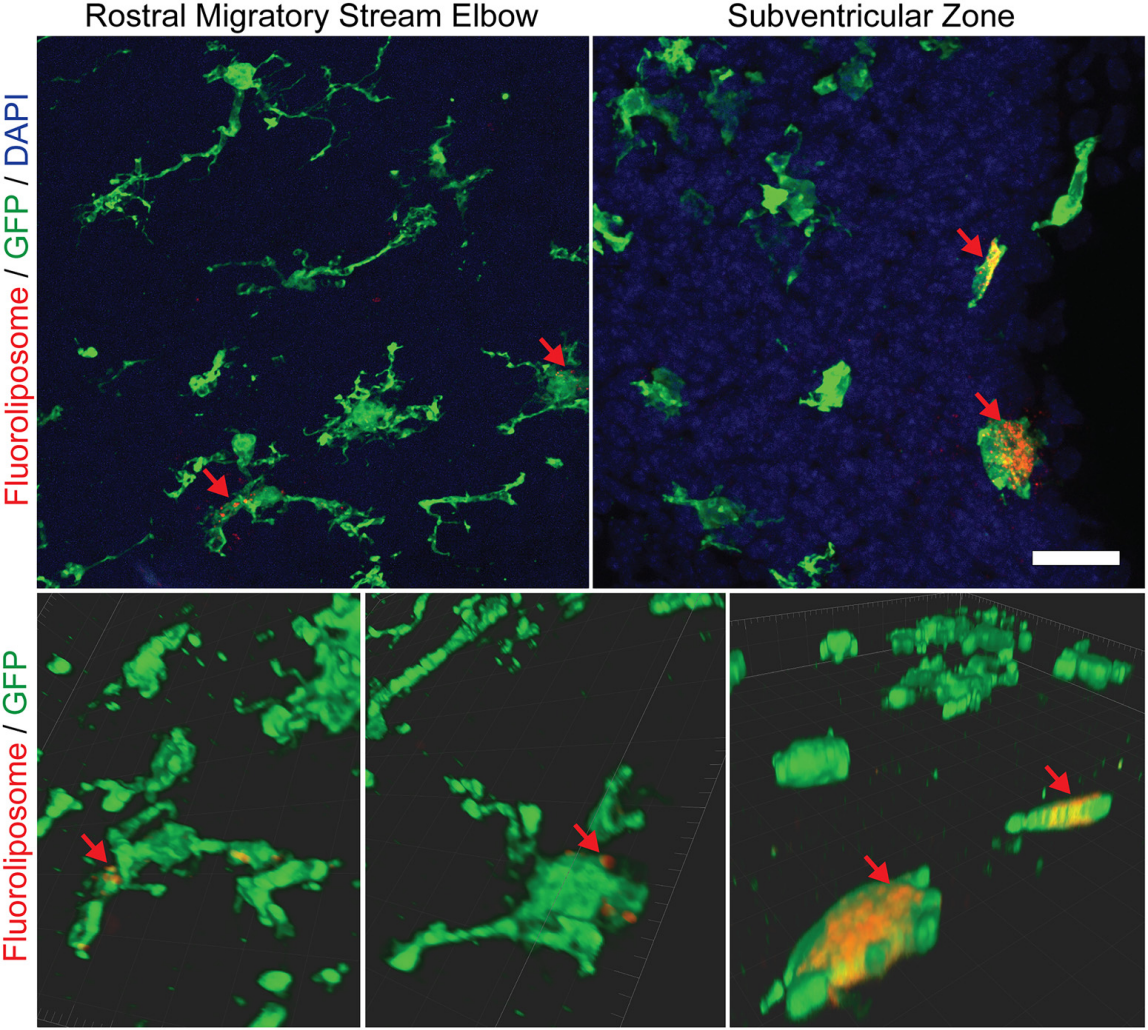
Fluoroliposomes accumulate within microglia in the subventricular zone (SVZ) and rostral migratory stream (RMS). The panels in the top row show sagittal sections of the SVZ and RMS elbow in *CX3CR-1^GFP/+^* mice at P4 after injection of fluoroliposome-Dil into the lateral ventricles at P1. Fluoroliposome-Dil have excitation/emission spectra of 550/565. The bottom row depicts 3D volume rendering of select microglia with Imaris software. Red arrows indicate fluoroliposomes accumulating within GFP+ microglia in the SVZ (right) and ventral border of the RMS (left). Scale bar = 25 µm.

We next sought to elucidate whether microglia monitor and phagocytose antigens in the RMS. To demonstrate the phagocytic capacity of microglia throughout the RMS, fluorescent liposomes containing the fluorescent dye Dil (Fluoroliposome) were injected into the cerebral lateral ventricles of P1 CX3CR-1^GFP/+^ mice and killed at P4. Fluoroliposomes accumulated within microglia to a high degree in the SVZ but were also observed in distal regions of the RMS (Fig. 8). Microglia thus appear to monitor and phagocytose extracellular material within the early postnatal RMS.

**Figure 9.**
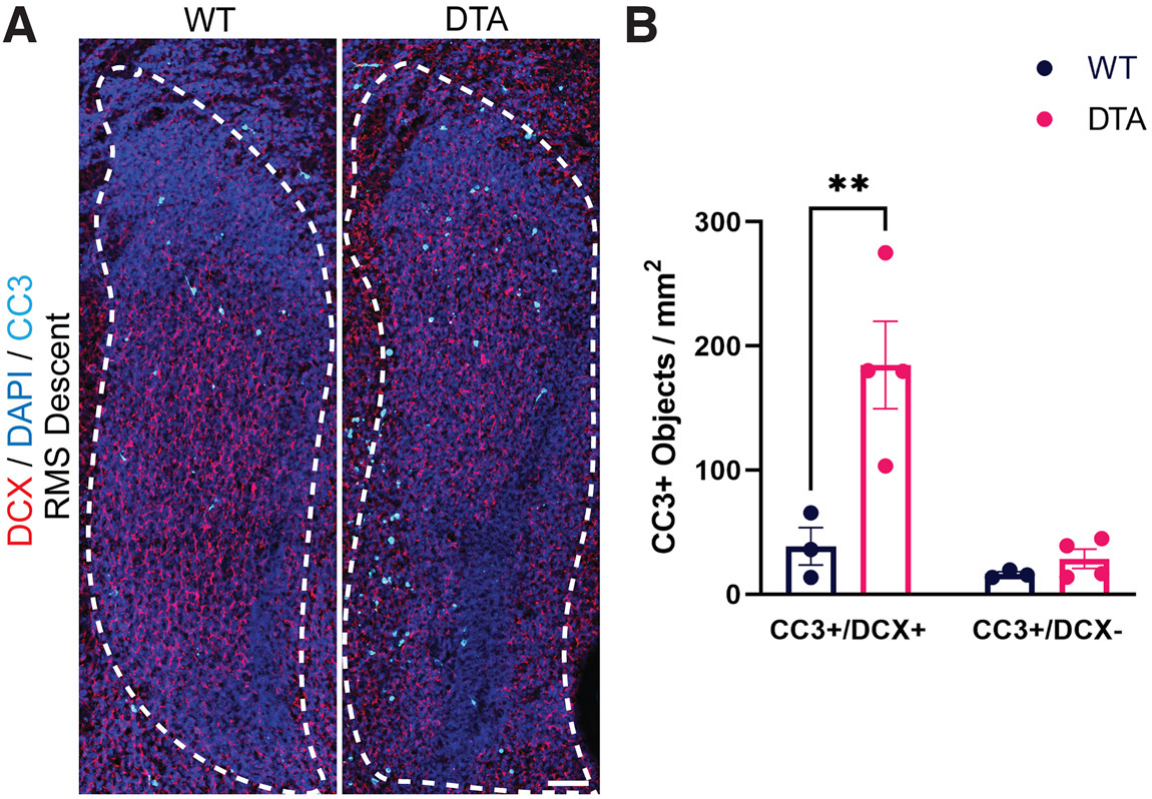
Microglia depletion for 3 d postnatally is associated with an accumulation of apoptotic cells in the rostral migratory stream (RMS). ***A***, Coronal sections of the descent of the RMS in *Cx3cr1^CreER/+^; ROSA26^+/+^* (left) and in *Cx3cr1^CreER/+^; ROSA26^eGFP-DTA/+^* (right) littermates following intraperitoneal injection of tamoxifen at postnatal day 0 (P0) and killing at P3. The RMS region was defined by increased density of DAPI nuclear staining, as shown by the white dashed outline. Apoptotic cells were identified with cleaved caspase-3 (CC3) labeling (cyan) and neuroblasts with doublecortin (DCX) staining (red). Scale bar = 50 µm**. *B***, There was a significant increase in the density of CC3+/DCX+ cells in the RMS of DTA+ mice following injection of tamoxifen in the RMS descent. There was no significant difference in the density of CC3+/DCX− cells in the RMS descent following microglia depletion. **p < 0.01.

**Figure 10.**
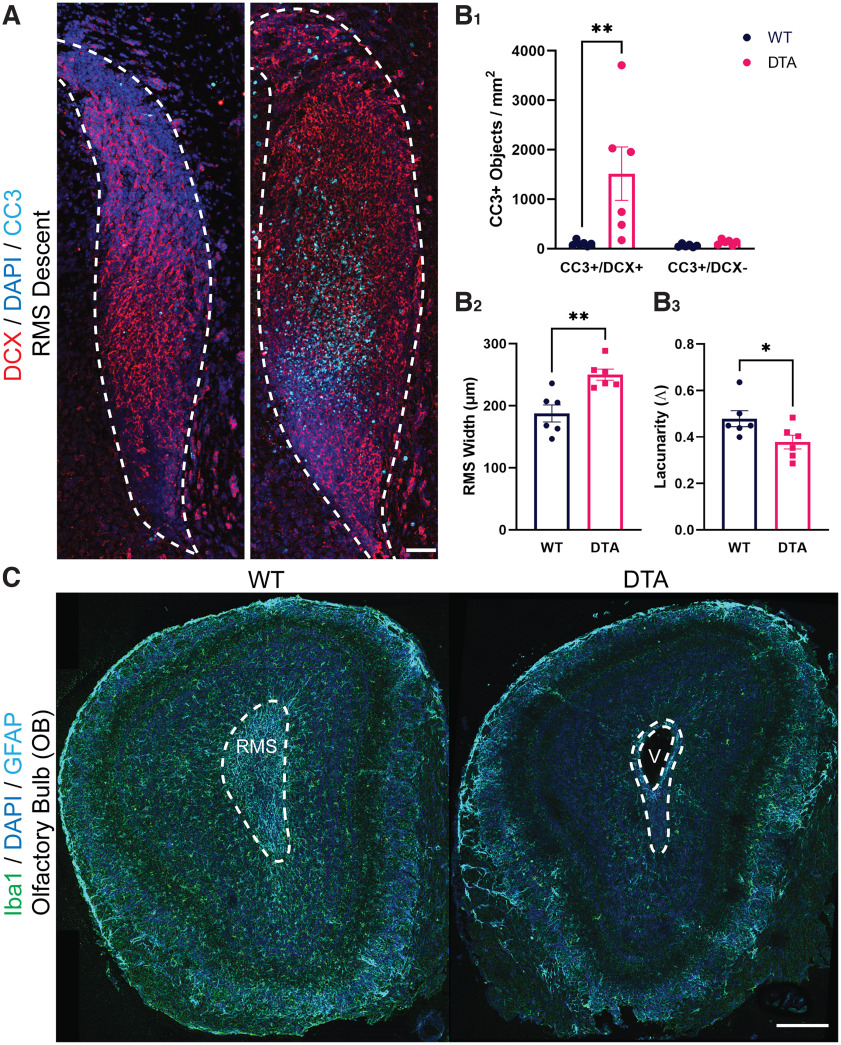
Microglia depletion for 14 d postnatally is associated with an accumulation of apoptotic cells and patent olfactory ventricles in the rostral migratory stream (RMS). ***A***, Coronal sections of the start and descent of the RMS in *Cx3cr1^CreER/+^; ROSA26^+/+^* (left) and in *Cx3cr1^CreER/+^; ROSA26^eGFP-DTA/+^* (right) littermates following intraperitoneal injection of tamoxifen at postnatal day 0 (P0) and every 3 d subsequently until killing at P14. Apoptotic cells were identified with cleaved caspase-3 (CC3) labeling (cyan) and neuroblasts with doublecortin (DCX) staining (red). Scale bar = 50 µm**. *B_1_***, There was a significant increase in the density of CC3+/DCX+ cells in the RMS of DTA mice following 14 d of microglia depletion in the RMS descent, but not in CC3+/DCX− cells. ***B_2_***, There was a significant increase in the width of the RMS at its widest point at the RMS descent. The RMS region was defined by increased density of DAPI nuclear staining, as shown by the wshite dashed outline in ***A***. ***B_3_***, There was a significant decrease in measures of lacunarity (Λ) of DCX+ stained processes in the RMS descent following microglia depletion, suggesting decreased gaps in the space between stained processes. ***C***, Coronal sections of the whole olfactory bulb (OB) in *Cx3cr1^CreER/+^; ROSA26^+/+^* (left) and in *Cx3cr1^CreER/+^; ROSA26^eGFP-DTA/+^* (right) littermates. Pups were injected every 3 d starting at P1 and killed at P14. The RMS region is defined by increased density of DAPI nuclear staining in the OB core, as shown by the white dashed outline. A decrease in the number of microglia (green) is seen in the OB of the DTA+ mouse (right) following TMX injections. Enlarged and patent olfactory ventricles were observed in DTA+ mice (V), as opposed to the closed olfactory ventricles of WT littermates (left). Scale bar = 250 µm. *p <0.05, **p < 0.01.

To demonstrate a functional role for microglia in regulating the population of migrating neuroblasts in the developing RMS, we depleted microglia for 3 and 14 d postnatally to evaluate whether there was an accumulation of apoptotic neuroblasts in the RMS. *Cx3cr1^CreER/+^; ROSA26^eGFP-DTA/+^* and *Cx3cr1^CreER/+^; ROSA26^+/+^* control littermates were injected with TMX intraperitoneally starting at P0 and killed at P3 or injected with TMX every 3 d subsequently until killing at P14. To label microglia, neuroblasts and apoptotic cells, immunohistochemistry was performed with primary antibodies against Iba1 (data not shown), doublecortin (DCX), and cleaved caspase-3 (CC3; Figs. 9*A*, 10*A*). There was a significant decrease in microglia density in *Cx3cr1^CreER/+^; ROSA26^eGFP-DTA/+^* animals compared with *Cx3cr1^CreER/+^; ROSA26^+/+^* control littermates after 3 d of TMX treatment (RMS start *p* < 0.0001; RMS descent *p* < 0.0001). Microglia depletion was associated with a significant increase in the accumulation of CC3+/DCX+ apoptotic cells in the RMS start and RMS descent at both P3 (WT vs DTA *p* < 0.01; Fig. 9*B*) and P14 (WT vs DTA *p* < 0.01; Fig. 10*B_1_*), suggesting an accumulation of apoptotic neuroblasts. There was no significant difference in the density of CC3+/DCX− apoptotic cells in the RMS following microglia depletion in either group (Fig. 10*B_1_*). An extended period of microglia depletion was also associated with an increase in width of the RMS descent (*p* < 0.01; Fig. 10*B_2_*) and decreased lacunarity of DCX-stained processes within the RMS (*p* < 0.05; Fig. 10*B_3_*). The expanded RMS width and decrease in “gappiness” between stained neuroblast processes may reflect an increase in the number of neuroblasts or neuroblast clumping within the RMS following microglia depletion. Of further interest, we also found patent olfactory ventricles in the core of the RMS in all six mice that expressed the DTA+ subunit following 14 d of microglia depletion (Fig. 10*C*).

## Discussion

We sought to examine whether the ameboid microglia that invade the RMS early in development regulate the population of migrating neuroblasts. We found that microglia line the outer borders of the RMS and intimately associate with migrating neuroblasts in the early postnatal period. Microglia depletion did not impact the migratory capacity of neuroblasts in the postnatal RMS. However, microglia express phagocytic and trophic markers, “wrap” migrating neuroblasts, and contain DsRed-labeled material within phagocytic cups, suggesting possible phagocytosis or trophic support of neuroblasts in the RMS. Furthermore, microglia depletion induced an accumulation of apoptotic and densely packed neuroblasts, an expanded RMS, and patent olfactory ventricles. Together, these results suggest that microglia function to maintain a healthy neuroblast population and environment permissive to neuroblast migration.

Microglia are present in the RMS during the first postnatal week, where they exhibit an ameboid morphology, collect at the borders of the RMS and closely associate with migrating neuroblasts. This microglia distribution occurs before the assembly of the vascular scaffold and astrocyte tube. There was only sparse CD31 blood vessel labeling in the RMS during the first postnatal week, which lengthened at P14 to resemble the scaffold that parallels the path of neuroblast migration in adults (Bozoyan et al., 2012). An astrocyte tube was similarly not observed during the first postnatal week. Instead, GFAP-stained radial glia radiated from the RMS core (Alves et al., 2002). However, neuroblast migration was orthogonal to these processes, suggesting that migration may be independent of radial glia (Alves et al., 2002). A fine network of astrocyte processes coalesced into thick septate processes at P21 that further consolidated into the densely packed astrocytic tube typical of adults at P56 (Peretto et al., 2005). The distribution of microglia flanking the outer borders of the RMS during the first postnatal week suggests that they may provide cues to migrating neuroblasts in the absence of the vascular scaffold and astrocyte tube.

This microglia cell distribution and morphology pattern suggests that microglia monitor the RMS environment and may apply trophic support or phagocytic functions when appropriate. There is a preponderance of ameboid microglia in the RMS during the first postnatal week (Xavier et al., 2015) before they shift to a more ramified morphology, indicating that microglia functions may differ between these periods. “Dense” and ameboid microglia in the RMS decreased in number by P14, with a concomitant increase in microglia process complexity by P21.

This change in microglia morphology could simply be a manifestation of normal microglial cell development. Microglial cell development follows a step-wise developmental program with distinct temporal stages in gene expression (Matcovitch-Natan et al., 2016; Hammond et al., 2019; Li et al., 2019; Masuda et al., 2019, 2020). Throughout microglial cell development there is a gradual expression of adult homeostatic genes, such as *Tmem119*, *P2ry12*, and *Hexb* (Bennett et al., 2016; Matcovitch-Natan et al., 2016). Meanwhile, mutations in the transcription factor SALL1 induce an abnormal ameboid microglia morphology and downregulation of the homeostatic genetic signature (Buttgereit et al., 2016; Utz et al., 2020). Alternatively, varying microglia morphologies and shifts in gene expression could reflect different functional activities of microglia at different developmental stages. While single-cell RNA-sequencing revealed little microglia transcriptomic heterogeneity in adult mice, microglia isolated from developing mice exhibited heterogeneous gene expression profiles, suggesting diverse functionality (Hammond et al., 2019; Li et al., 2019; Masuda et al., 2019). Postnatal microglia transcript clusters further illustrated varied distribution across brain locations, implicating local specification of microglia function during development (Masuda et al., 2019). Meanwhile, whole genome transcriptional profiling of microglia showed enrichment of biological processes related to neurogenesis and neural development during embryonic life (E14.5) and early postnatal life (P1), but not adulthood (Grassivaro et al., 2020). Thus, specific microglia subsets may occur in a spatially and temporally restricted matter to regulate specific neurodevelopmental events.

We next considered whether the ameboid microglia lining the RMS in the first postnatal week regulate the population of migrating neuroblasts. Microglia do not appear to alter the intrinsic migratory capacity of neuroblasts in the RMS: microglia depletion for 3 d postnatally with two different depletion strategies did not alter the density of neuroblasts within the RMS nor the distance they migrated. These results indicate that microglia are not necessary for neuroblast migration in the early postnatal RMS. This is consistent with the findings of Kyle et al., who found no difference in the density of BrdU-labeled neuroblasts 4 d after BrdU treatment in the RMS of adult animals treated with the oral CSF-1R inhibitor PLX5622 (Kyle et al., 2019). However, microglia depletion for 14 d postnatally did induce an accumulation of apoptotic neuroblasts and decreased lacunarity of DCX-stained neuroblast processes within a broader RMS domain, suggests clumping of apoptotic neuroblasts to a degree that widens the RMS. These findings are consistent with the buildup of apoptotic cells observed after microglia depletion and selective microglial MERTK knock-down in the RMS of adult animals (Ribeiro Xavier et al., 2015; Fourgeaud et al., 2016).

The accumulation of apoptotic neuroblasts following microglia depletion could be secondary to a lack of appropriate microglia phagocytosis of defective or apoptotic neuroblasts. Microglia lining the RMS in the first postnatal week express the phagocytic markers CLEC7A, MERTK, and CD68; in contrast, microglia in the cortical regions outside the RMS do not, suggesting a specific role for microglia phagocytosis in the RMS. Microglia further contain DsRed-labeled neuroblast material within phagocytic cups. This parallels the findings of P7 postnatal microglia that express the phagocytic marker CLEC7A in the corpus callosum (Li et al., 2019). These microglia demonstrated an increase phagocytic capacity, engulfing fluorescent beads in brain slices (Li et al., 2019). These microglia also contained CC3 positive inclusions that were positive for myelin basic protein, suggesting that microglia phagocytose oligodendrocytes. Interestingly, oligodendrocytes that were negative for cleaved caspase three were also frequently observed to be “hugged” by early postnatal microglia, suggesting that they may actively contribute to eliminating oligodendrocytes in developing white matter tracts (Li et al., 2019). Similarly, we found that microglia expressing the phagocytic markers CLEC7A, CD68, and MERTK “hug” neuroblasts in the RMS, potentially suggesting phagocytosis of migrating neuroblasts.

The accumulation of neuroblasts within a wider RMS following microglia depletion suggests that a surplus of neuroblasts may be generated during development, which are subsequently eliminated by microglia. Microglia phagocytose neural precursor cells in both the rat and primate SVZ during all stages of prenatal development (Cunningham et al., 2013) as well as maturing neurons in the OB (Lazarini et al., 2012; Denizet et al., 2017). Microglia can also “multitask,” with individual cells simultaneously enveloping and phagocytosing multiple neural precursor cells (Barger et al., 2019). A role of microglia for clearing defective cells via phagocytosis may be reflected in the child born without microglia (Oosterhof et al., 2019); the observed heterotopias may be a result of a lack of appropriate phagocytic clearance of defective neuroblasts, as opposed to a general failure of neuroblast migration in the absence of microglia. However, it remains unknown whether microglia simply phagocytose dead or dying neuroblasts in the RMS, or whether they “kill” neuroblasts in the RMS via phagoptosis. There is evidence that microglia can “kill” cells in neurogenic regions through an extracellular respiratory burst (Marín-Teva et al., 2004; Sierra et al., 2010). Microglia depletion caused an increase in Purkinje cell number in the cerebellum, suggesting that microglia eliminated “living” and not apoptotic Purkinje cells (Marín-Teva et al., 2004). Microglia also phagocytosed mitotically active neural precursor cells independent of apoptotic pathways in the SVZ (Cunningham et al., 2013). Furthermore, despite the accumulation of apoptotic cells in the RMS of *Axl* and *Mertk* knock-out animals, there was a ∼70% increase in the cellular density of the granule cell and glomerular layers of the OB, suggesting that many neuroblasts phagocytosed by microglia are not apoptotic but instead “eaten-alive” (Fourgeaud et al., 2016). Future studies may reveal whether microglia randomly target migrating neuroblasts for phagoptosis or are able to detect aberrantly migrating or otherwise defective neuroblasts.

Alternatively, the accumulation of apoptotic neuroblasts following microglia depletion and microglia “wrapping” of neuroblasts could suggest trophic support of migrating neuroblasts. IGF-1 is also expressed by the CD68+ CLEC7A+ microglia subtype in the first postnatal week (Hammond et al., 2019; Li et al., 2019) and supports oligodendrocyte precursor cells (Wlodarczyk et al., 2017) and projecting axons of layer V cortical neurons (Ueno et al., 2013) in the corpus callosum. We also observed IGF-1 expression in RMS microglia during the first postnatal week. Thus, withdrawal of supportive trophic factors, such as IGF-1, following microglia depletion could result in the accumulation of apoptotic neuroblasts that we observed in the RMS. There may also be regional heterogeneity of microglia functions within the RMS, with some microglia being responsible for trophic support and others for neuroblast elimination. Such heterogeneity was demonstrated in ameboid microglia expressing SPP1 in the corpus callosum during the first postnatal week: microglia expressing high transcript levels of *Spp1* neighbored microglia with no expression (Hammond et al., 2019). Paralleling this finding, SPP1 was also observed in a subset of CLEC7A+ microglia in the developing corpus callosum of P7 adjacent to CLEC7A+ with no SPP1 expression (Li et al., 2019). Similarly, we observed microglia with high transcript levels of *Igf1* neighboring microglia with no expression in the RMS start. There may therefore be local heterogeneity of microglia functions in the early postnatal RMS, with microglia responsible for neuroblast elimination neighboring microglia that support neuroblast survival.

Microglia also phagocytose labeled antigens in distal regions of the RMS, indicating the monitoring and phagocytosis of extracellular material. The enlarged and patent olfactory ventricles we observed following 14 d of postnatal microglia depletion could also be secondary to deficient microglia phagocytosis. It is possible that phagocytic microglia lining the RMS are responsible for clearing apoptotic debris, and thus an accumulation of debris in their absence may obstruct normal CSF outflow, increase CSF pressure and induce ventriculomegaly. This parallels early findings of enlarged and patent olfactory ventricles observed in three-week-old *Csf1* null mice that have a significant depletion of microglia (Erblich et al., 2011). An accumulation of apoptotic debris and other extracellular material in the RMS may similarly impede neuroblast migration over time. Thus, another important function of microglia in the developing RMS may be the monitoring of draining antigens and maintenance of RMS homeostasis by responding to these antigens and clearing debris.

In summary, our data suggest that a specialized microglia subtype is involved in maintaining a healthy neuroblast population and environment permissive to neuroblast migration in the RMS during early postnatal development. We found that ameboid microglia closely associate with migrating neuroblasts in the postnatal RMS before the formation of the glial tube and vascular scaffold that facilitate neuroblast migration in adulthood. Microglia in the first postnatal week further express the markers CD68, MERTK, CLEC7A, and IGF-1 and “wrap” migrating neuroblasts within the RMS. While the migratory capacity of neuroblasts was unaffected by microglia depletion, extended microglia depletion induced an accumulation of apoptotic neuroblasts within a broader migratory corridor. Our data suggest that a specialized ameboid microglia subtype expressing the markers CD68, MERTK, CLEC7A, and IGF-1 is critical for maintaining a healthy neuroblast population that enables an environment permissive to neuroblast migration in the RMS. As our understanding of the functional capabilities of microglia continues to expand, these results shed important insight into the role of microglia in regulating the homeostasis of neuroblast migratory corridors during the early postnatal period.

## Synthesis

Reviewing Editor: Matthew Grubb, King’s College London Decisions are customarily a result of the Reviewing Editor and the peer reviewers coming together and discussing their recommendations until a consensus is reached. When revisions are invited, a fact-based synthesis statement explaining their decision and outlining what is needed to prepare a revision will be listed below. The following reviewer(s) agreed to reveal their identity: Isabelle Caillé.

Reviewer 1:

Advances the Field:

While the roles of microglia in the maintenance of neural stem/progenitor cells and synaptic remodeling of mature neurons have been intensively studied, those in neuronal migration in the postnatal brain remain unexplored. This manuscript has demonstrated the importance of microglia in neuronal migration in the postnatal brain, which provides significant advances in the field of microglial biology and postnatal neurogenesis.

Comments to the Authors:

In this study, the authors examined the contribution of microglia in the neuroblast migration in the neonatal RMS. Amoeboid microglia assemble along the outer borders of the RMS and tightly associate with neuroblasts. Microglial depletion experiments revealed that microglia contribute to the maintenance of cells and structure in the neonatal RMS. Overall, the subject of this study is very interesting and the authors’ findings provide significant advances in the fields of microglia and postnatal neurogenesis. However, the authors’ conclusion about the role of microglial phagocytosis of neuroblasts in the homeostasis of neuroblast migration is not fully supported by sufficient experimental evidence and proper interpretations in the current version of the manuscript. Addressing the following comments will improve the study and manuscript.

Major comments

１） Fig. 9: Although the authors conclude that microglia phagocytose neuroblasts in the developing RMS based on microglial marker expressions and wrapping morphology, no experimental evidence showing microglial phagocytosis of neuroblasts (i.e. images for neuroblasts completely incorporated in microglial processes or soma) was shown. Since microglia reversibly wrap and retract their membrane along their targets, wrapping could be an initial step for phagocytosis but not identical to phagocytosis. Therefore, please show representative three-dimensional confocal images for microglial phagocytosis of neuroblasts with quantification. Alternatively, please reconsider the interpretation of wrapping; wrapping could be involved in not only phagocytosis but also trophic support.

２） Figs. 11 and 12: Microglial ablation experiments could not specifically address the role of microglial phagocytosis (page 17, line 10∼) because microglia have various biological functions. Even if the authors could observe microglial phagocytosis of neuroblasts in the developing RMS, the purpose and interpretation of microglial ablation experiments should be described and discussed carefully throughout their manuscript.

３） Figs. 11 and 12: Because DTA mice eliminate microglia following tamoxifen injection, cleaved caspase-3+ cells in the neonatal RMS could be composed of not only neuroblasts but also microglia. In Figs. 11B2 and 12B2, could you assess the number of Dcx+ cleaved caspase-3+ cells to support the authors’ conclusion?

４） In Fig. 8, BrdU single-positive cells do not simply reflect neuroblasts, since BrdU could be incorporated in both neuroblasts and glial cells in the neonatal stage. Please count Dcx+ BrdU+ cells observed in the glomerular layer.

Minor comments

５） Fig. 1: This figure does not include any novel methods. Please consider including these illustrations in other figures (for instance, Fig 1A to Fig 6, and Fig. 1B to 7/11/12).

６） Fig. 2C1, 2C2: Nuclei of green-labeled microglia seem to be pyknotic morphologies at current low resolution. Please show more representative images of microglia with living nuclei (like Fig. 2C3 and 2C4) or magnify current images to clearly visualize nuclear features.

７） Fig. 9B-D: Marker expressions in GFP+ microglia could not be visible in merged images. Please show each channel image in Fig. 9B, C, and D to clearly see CD68, CLEC7A, and MERTK signals.

８） Fig. 7C1 and 11B1: These might be redundant data with different control values.

９） Methods: microglia “knockdown” (page 6, line 2, line 23, etc) should be revised as microglia “depletion” or other appropriate words.

Reviewer 2:

Advances the Field:

The manuscript nicely describes microglia morphology and distribution in the postnatal Rostral Migratory Stream and analyze their potential role in migration. This is new and interesting in the field

Comments to the Authors:

This interesting and elegant work describes microglial cells in the postnatal RMS and analyze their potential function. By combining pharmacological and genetic approaches, the authors conclude that microglia do not play a direct role in tangential migration but probably in the reorientation of neuroblasts in the olfactory role. They also describe active phagocytosis of neuroblasts by microglia leading to a wider RMS in microglia-depleted animals.

The overall work is nicely performed and convincing as well as very well written.

I would argue, however, that the authors should perform one or two additional experiments before coming to the (rather disapointing) conclusion that neuroblast migratory capacity is independent of microglia.

In the clodronate-filled liposomes experiment, they concomitantly inject BrdU and clodronate in P0 animals to assess migration at P3. In the genetic ablation model, similarly, they inject tamoxifen and electroporate mice at P0, to assess migration at P3. I think that one or both experiments should be performed with a delay between microglia depletion and labeling of neuroblasts. One interpretation of the lack of effect could be that neuroblasts have the time to migrate before actual microglia depletion. Indeed, the fact that they do observe a migration defect when analyzing migration later in the olfactory bulb might stem from a larger delay for neuroblasts ro reach hte OB.

I am thus looking forward to knowing the result of this experiment, which might unveil a role for microglia in regulating RMS migration !

## Author Response

First and foremost, thank you so much for the supportive and encouraging reviews of our ms. entitled “Microglia maintain homeostatic conditions in the developing rostral migratory stream.” We very much appreciate the enthusiasm of the reviewers and the very helpful and constructive suggestions they have provided. We have carefully followed all of their recommendations and revised the ms. accordingly. Throughout the main text and figure captions revisions/edits are identified with highlighting.

Second, I also will take this opportunity to thank you for the extension on the submission of this revision.

Synthesis of Reviews:

Three primary points are summarized that include 1) addressing more thoroughly neuroblast migration after microglia depletion; 2) examples of microglia phagocytosing neuroblasts; and 3) consideration of alternative interpretations of the data. Each of these are addressed in detail below for each of the reviewers.

Reviewer #1

Thank you so much for the supportive comment - “This manuscript has demonstrated the importance of microglia in neuronal migration in the postnatal brain, which provides significant advances in the field of microglial biology and postnatal neurogenesis.” We are very excited about these studies and believe that they may also have broader implications for neuroblast migration elsewhere in the nervous system.

Overarching comment - “...the authors’ conclusion about the role of microglial phagocytosis of neuroblasts in the homeostasis of neuroblast migration is not fully supported by sufficient experimental evidence and proper interpretations in the current version of the manuscript. Addressing the following comments will improve the study and manuscript.” We do so below.

Major Comments

1) ”...no experimental evidence showing microglial phagocytosis of neuroblasts (i.e. images for neuroblasts completely incorporated in microglial processes or soma) was shown .... please show representative three-dimensional confocal images for microglial phagocytosis of neuroblasts with quantification. Alternatively, please reconsider the interpretation of wrapping; wrapping could be involved in not only phagocytosis but also trophic support.”

RESPONSE - Thank you for this constructive suggestion. To further support our interpretation of phagocytosis we have included additional experimental evidence using 3D imaging illustrating neuroblast material within phagocytic cups (see Fig. 7B). However, we also agree with the reviewer that an alternative interpretation may be that microglia provide trophic support to neuroblasts within the RMS. Thus, we have carefully revised our reporting of the data and the discussion to include trophic support of neuroblasts as well as phagocytosis.

We argue that RMS microglia in the first postnatal week express the markers CLEC7A, CD68 and IGF-1 and may be involved in either trophic support or phagocytosis of neuroblasts to support a healthy neuroblast population; this could be a population similar to the ameboid microglia that express these markers in developing white matter tracts during the first postnatal week and are involved in regulating oligodendrocyte precursor cells (Hagemeyer et al., 2017; Hammond et al., 2019; Li et al., 2019).

We have revised the manuscript as follows to support this argument:

- The introduction of the paper was reorganized to highlight our interest in this microglia subtype.

- Under Results, the header “Microglia phagocytosis in the developing rostral migratory stream” has been changed to “Microglia mediate the homeostasis of the developing rostral migratory stream.”

- Under Results we wrote “We capitalized on these recent findings to ask if microglia in the RMS the phagocytic markers CLEC7A, MERTK, CD68 (Fig. 7A) and trophic factor IGF-1 during early postnatal development.”

- We included additional analysis using fluorescence in situ hybridization to evaluate transcript levels of IGF-1 in microglia of P4 mice. We did this to provide further evidence that microglia in the early postnatal RMS may be comprised of the specialized microglia subtype identified by recent single-cell sequencing studies (Hagemeyer et al., 2017; Hammond et al., 2019; Li et al., 2019), as well as to support the possibility that microglia provide trophic support to migrating neuroblasts.

- We included the following in the results: “Microglia in the RMS start of P4 CX3CR-1GFP/+ mice expressed high transcript levels of Igf1; however, there was heterogeneity of expression, with microglia expressing high transcript levels of Igf1 immediately adjacent to microglia with no appreciable expression (Fig. 7G).”

2) ”...Microglial ablation experiments could not specifically address the role of microglial phagocytosis (page 17, line 10∼) because microglia have various biological functions. Even if the authors could observe microglial phagocytosis of neuroblasts in the developing RMS, the purpose and interpretation of microglial ablation experiments should be described and discussed carefully throughout their manuscript.”

RESPONSE - Thank you for this astute comment. We agree that we did not address the broad array of microglia biologic functions that could be implicated in the phenotype we observed following microglia depletion. We altered the manuscript throughout to be more careful of our interpretations. We altered our Discussion as follows:

- We re-wrote the first paragraph of the Discussion as follows in order to be more circumspect regarding the conclusion that microglia regulate the RMS solely through phagocytic functions:

"However, microglia express phagocytic and trophic markers, “wrap” migrating neuroblasts, and contain DsRed-labeled material within phagocytic cups, suggesting possible phagocytosis or trophic support of neuroblasts in the RMS. Furthermore, microglia depletion induced an accumulation of apoptotic and densely packed neuroblasts, an expanded RMS, and patent olfactory ventricles. Together, these results suggest that microglia function to maintain a healthy neuroblast population and environment permissive to neuroblast migration.”

- While we retained a discussion of how the accumulation of apoptotic neuroblasts could be secondary to a lack of appropriate microglia phagocytosis, we included the alternative interpretation as well:

"Alternatively, the accumulation of apoptotic neuroblasts following microglia depletion and microglia “wrapping” of neuroblasts could suggest trophic support of migrating neuroblasts. IGF-1 is also expressed by the CD68+ CLEC7A+ microglia subtype in the first postnatal week (Hammond et al., 2019; Li et al., 2019a) and supports oligodendrocyte precursor cells (Wlodarczyk et al., 2017) and projecting axons of layer V cortical neurons (Ueno et al., 2013) in the corpus callosum. We also observed IGF-1 expression in RMS microglia during the first postnatal week. Thus, withdrawal of supportive trophic factors, such as IGF-1, following microglia depletion could result in the accumulation of apoptotic neuroblasts that we observed in the RMS. ... Similarly, we observed microglia with high transcript levels of Igf1 neighboring microglia with no expression in the RMS start. There may therefore be local heterogeneity of microglia functions in the early postnatal RMS, with microglia responsible for neuroblast elimination neighboring microglia that support neuroblast survival.”

- We removed our conclusion that microglia regulate neuroblasts through phagocytosis in the concluding paragraph of our discussion. The revision now reads:

"In summary, our data suggest that a specialized microglia subtype is involved in maintaining a healthy neuroblast population and environment permissive to neuroblast migration in the RMS during early postnatal development. ... Microglia in the first postnatal week further express the markers CD68, MERTK, CLEC7A and IGF-1 and “wrap” migrating neuroblasts within the RMS. While the migratory capacity of neuroblasts was unaffected by microglia depletion, extended microglia depletion induced an accumulation of apoptotic neuroblasts within a broader migratory corridor. Our data suggest that a specialized ameboid microglia subtype expressing the markers CD68, MERTK, CLEC7A and IGF-1 is critical for maintaining a healthy neuroblast population that enables an environment permissive to neuroblast migration in the RMS. As our understanding of the functional capabilities of microglia continues to expand, these results shed important insight into the role of microglia in regulating the homeostasis of neuroblast migratory corridors during the early postnatal period.

- We changed the title of Figure 7 to “Specialized microglia subtype in the postnatal rostral migratory stream (RMS).”

3) ”...cleaved caspase-3+ cells in the neonatal RMS could be composed of not only neuroblasts but also microglia. In Figs. 11B2 and 12B2, could you assess the number of Dcx+ cleaved caspase-3+ cells to support the authors’ conclusion?"

RESPONSE - Thank you for this excellent suggestion. We altered the analysis to assess the number of DCX+ cleaved caspase 3+ cells in the RMS after both 3 and 14 days of microglia depletion, as suggested by the reviewer (see Fig. 9B and 10B1).

4) "In [former] Fig. 8, BrdU single-positive cells do not simply reflect neuroblasts, since BrdU could be incorporated in both neuroblasts and glial cells in the neonatal stage. Please count Dcx+ BrdU+ cells observed in the glomerular layer.”

RESPONSE - This was a good point and we have followed through with the suggestion in the RMS. We have eliminated the former Figure 8 and analysis of the glomerular layer in order to focus on our primary area of interest, the RMS. See revised text discussion of labeling of cells other than neuroblasts with the single-pulse BrdU.

Minor Comments

5) "Figure 1 does not include any novel methods"

RESPONSE - We have removed the original Figure 1 as recommended.

6) "Fig. 2C1, 2C2: Nuclei of green-labeled microglia seem to be pyknotic morphologies at current low resolution. Please show more representative images...”

RESPONSE - Great catch and an oversight on our part. We have now included two new electron micrographs (Fig. 1 C1 and C2) that are prototypical for microgial including the accumulation of chromatin on the inner membrane of the nucleus, the presence of lipofuscin granules in the cytoplasm and their overall smaller size relative to neuroblasts. See new discussion in Figure 1 caption and in the Results section.

7) "[former] Fig. 9B-D: Marker expressions in GFP+ microglia could not be visible in merged images. Please show each channel image in Fig. 9B, C, and D to clearly see CD68, CLEC7A, and MERTK signals.”

RESPONSE - We have revised the figure (now Fig. 7) extensively and show each channel separately as well as in the merged format.

8) "Fig. 7C1 and 11B1: These might be redundant data with different control values.”

RESPONSE - While these were separate experiments performed with different animals, it is true that the method is the same and thus the main finding (that microglia depletion is successfully accomplished when tamoxifen is injected in Cx3cr1CreER/+; ROSA26eGFP-DTA/+ mice) is redundant. Thus we removed this data from Fig. 9 and provided the p value of the student t-test examining the difference in microglia density following tamoxifen injection in the body of the text.

9) "Methods: microglia “knockdown” (page 6, line 2, line 23, etc) should be revised as microglia “depletion” or other appropriate words.”

RESPONSE - A very appropriate suggestion and we have accordingly substituted “depletion” for “knockdown” throughout the manuscript. A much more accurate description as suggested by the reviewer.

Reviewer #2

We very much appreciate the observation by Reviewer #2 that: “This [finding] is new and interesting in the field” and that “This interesting and elegant work describes microglial cells in the postnatal RMS and analyze their potential function...

The overall work is nicely performed and convincing as well as very well written.”

Comments to Authors

"I would argue, however, that the authors should perform one or two additional experiments before coming to the (rather disappointing) conclusion that neuroblast migratory capacity is independent of microglia...experiments should be performed with a delay between microglia depletion and labeling of neuroblasts...[because]...the lack of effect could be that neuroblasts have the time to migrate before actual microglia depletion.”

RESPONSE - This is indeed an interesting and important observation and one to which we have continued to give consideration. First, we now include a citation to an earlier paper (Kyle et al. 2019) who also reported that depletion of microglia in adult mice did not affect neuroblast migration in the RMS. However, to re-examine this issue more thoroughly in our own data we have carried out an additional experiment to assess lacunarity of DCX stained neuroblasts in the RMS after a period of 14 continuous days of depletion of microglia. As we previously reported, after 3 days of depletion we found no evidence of neuroblast density within the RMS. However, after 14 days the lacunarity of DCX-stained processes decreased significantly in the RMS, suggesting a decrease in gaps between stained processes and neuroblast “clumping.” (Figure 10B3). There was a corresponding increase in the dimensions of the RMS due to the accumulation of the apoptotic neuroblasts (Figure 10B2). These data suggest the plausible interpretation that any changes in the efficacy of neuroblast migration in the RMS are due to the accumulation of apoptotic cells or debris that then compromise the migratory route.

We report this new finding under Results as follows:

"An extended period of microglia depletion was also associated with an increase in width of the RMS descent (p < 0.01; Fig. 10B2) and decreased lacunarity of DCX-stained processes within the RMS (p < 0.05; Fig. 10B3). The expanded RMS width and decrease in “gappiness” between stained neuroblast processes may reflect an increase in the number of neuroblasts or neuroblast clumping within the RMS following microglia depletion.”

And in the legend of Figure 10 as follows: “B3) There was a significant decrease in measures of lacunarity (Λ) of DCX+ stained processes in the RMS descent following microglia depletion, suggesting decreased gaps in the space between stained processes.”

Also from Reviewer #2

"I am thus looking forward to knowing the result of this experiment, which might unveil a role for microglia in regulating RMS migration!"

RESPONSE - Well, we too had hypothesized a more profound and direct effect on neuroblast migration when we depleted the microglia. However, that should not diminish the impact of our observations on the role of microglia in the RMS for maintaining a homeostatic environment, plausibly in part through the phagocytosis of apoptotic cells or debris or through trophic support of migrating neuroblasts.

Once again thank you for your support and patience.
